# Identification and Characterization of Differentially Expressed Genes in Inferior and Superior Spikelets of Rice Cultivars with Contrasting Panicle-Compactness and Grain-Filling Properties

**DOI:** 10.1371/journal.pone.0145749

**Published:** 2015-12-28

**Authors:** Sudhanshu Sekhar, Sachin Ashruba Gharat, Binay Bhushan Panda, Trupti Mohaptra, Kaushik Das, Ekamber Kariali, Pravat Kumar Mohapatra, Birendra Prasad Shaw

**Affiliations:** 1 Environmental Biotechnology Laboratory, Institute of Life Sciences, Bhubaneswar, Odisha, India; 2 School of Life Sciences, Sambalpur University, Jyoti Vihar, Sambalpur, Odisha, India; National Institute of Plant Genome Research, INDIA

## Abstract

Breeding programs for increasing spikelet number in rice have resulted in compactness of the panicle, accompanied by poor grain filling in inferior spikelets. Although the inefficient utilization of assimilate has been indicated as responsible for this poor grain filling, the underlying cause remains elusive. The current study utilized the suppression subtractive hybridization technique to identify 57 and 79 genes that overexpressed in the superior and inferior spikelets (with respect to each other), respectively, of the compact-panicle rice cultivar Mahalaxmi. Functional categorization of these differentially expressed genes revealed a marked metabolic difference between the spikelets according to their spatial location on the panicle. The expression of genes encoding seed storage proteins was dominant in inferior spikelets, whereas genes encoding regulatory proteins, such as serine-threonine kinase, zinc finger protein and E3 ligase, were highly expressed in superior spikelets. The expression patterns of these genes in the inferior and superior spikelets of Mahalaxmi were similar to those observed in another compact-panicle cultivar, OR-1918, but differed from those obtained in two lax-panicle cultivars, Upahar and Lalat. The results first suggest that the regulatory proteins abundantly expressed in the superior spikelets of compact-panicle cultivars and in both the superior and inferior spikelets of lax-panicle cultivars but poorly expressed in the inferior spikelets of compact-panicle cultivars promote grain filling. Second, the high expression of seed-storage proteins observed in the inferior spikelets of compact-panicle cultivars appears to inhibit the grain filling process. Third, the low expression of enzymes of the Krebs cycle in inferior spikelets compared with superior spikelets of compact-panicle cultivars is bound to lead to poor ATP generation in the former and consequently limit starch biosynthesis, an ATP-consuming process, resulting in poor grain filling.

## Introduction

Rice is a staple food for the majority of the worldwide population, accounting for nearly 23% of the human consumption of carbohydrates in the form of cereals [1]. In addition, it has been estimated that world rice production must increase to at least 800 million tons from the current production of 585 million tons to account for the rapidly increasing global population [2], which is likely to increase from 7.3 billion [3] to 9.6 billion by 2050 [4]. Breeding efforts to increase the yield potential of rice have increased the number of spikelets per panicle, expanding the yield sink capacity (the maximum size of sink organs to be harvested), such as the NPT (new plant type) of IRRI and ‘super’ rice or ‘super’ hybrid rice in China [5,6]. However, this development was accompanied by an increase in panicle compactness as well as poor grain filling and unfilled grains, thus restricting grain yield [5,7–9], which is a product of both yield sink capacity and filling efficiency.

Khush and Peng [10] reviewed the yield performance of NPT lines and hypothesized that increases in the number of spikelets per panicle will result in disadvantages to the spikelets in the lower portion of the panicle in terms of carbohydrate availability and grain filling; this is potentially because the additional spikelets are primarily located on secondary branches of the panicle [11]. Through several spikelet-removal treatments immediately after heading, Kato [12] demonstrated that poor grain filling in spikelets on secondary branches is largely due to source-limited conditions, likely at specific stages of grain filling. However, it has also been observed that the synthesis of starch in the endosperm cells of spikelets on secondary branches is poor [13] and that the assimilates partitioned to these cells remain unused [8], suggesting that the sink rather than the source may be responsible for the observed effect in the transport and storage of assimilates [8,9]. This possibility is also reflected in the findings that the grain-filling rate of several rice cultivars, including NPT lines, is not associated with light-saturated photosynthesis [2] and that low activity and/or gene expression levels of starch-biosynthesis-related enzymes is associated with poor grain filling in basal spikelets [6,9]. However, the basic mechanism regulating this process remains elusive because the removal of some of the primary branches from the axis improves the grain-filling percentage compared with that obtained in uncut panicles [14].

The possibility that ethylene influences the grain-filling process in rice has also been suggested because the use of an ethylene inhibitor improves the growth and development of the inferior spikelets on basal branches of the panicle, whereas the application of an ethylene promoter inhibits the growth and development of these spikelets [15]. Moreover, the ethylene evolution rate is significantly negatively correlated with cell division and grain-filling rates [16], and hormones inhibit the activities of starch-synthesizing enzymes, thereby decreasing assimilates partitioning to the developing caryopsis [8,17]. Furthermore, inferior spikelets produce more ethylene than superior spikelets [8,16], and the compact spikelet arrangement in the panicle axis increases ethylene production to the detriment of grain filling [8]. Regardless, participation of ethylene in the regulation of inter-grain signaling is a comparatively recent addition to the role of hormones in rice-grain development. The present study exploits differences between various rice cultivars in terms of panicle compactness and ethylene evolution in superior (apical) and inferior (basal) spikelets to determine the possible causes of the observed differences in grain filling. We applied a comparative analysis of differentially expressed genes in these spikelets during the initial period of grain filling using the PCR-based suppression subtractive hybridization (SSH) technique. The findings indicate that high accumulation of seed storage proteins during the early period post-anthesis and inefficient active transport of assimilates from the nucellus to the aleurone layer and endosperm cells are the most probable reasons for the poor grain filling observed in inferior spikelets.

## Materials and Methods

### Rice cultivars and experimental site

Seeds from several *Oryza sativa* indica cultivars with different maturity durations, including Satyakrishna, Upahar, Lalat, OR-1918, Ratna, Sebati, Jaya, Manika, TJ-112 and Mahalaxmi, were screened for spikelet ethylene evolution, panicle compactness and the size and number of spikelets per panicle during the Kharif season in the years 2011 and 2012. The plants were grown on private farmland at the outskirts of Bhubaneswar city in Orissa, India, with permission from the owner. The plants were grown following standard agricultural practices for seed sowing, transplantation, irrigation, fertilizer use and pesticide application. The field studies did not involve endangered or protected species. Based on data for panicle morphology (S1 Table) and spikelet ethylene evolution (S1 Fig), four cultivars of *O*. *sativa* indica, two with a long maturity duration (Mahalaxmi and Upahar) and two with a medium maturity duration (OR-1918 and Lalat), were considered for the subsequent experiments performed in this study. These cultivars were grown at the Central Rice Research Institute (CRRI), Cuttack, India, during the subsequent Kharif seasons in the years 2013 and 2014 for the collection of samples for analysis, as described below. The field facility at CRRI was used with the permission of the Director of the organization.

### Sampling and harvesting

All of the primary tillers of a cultivar showing anthesis in apical (superior) spikelets in the morning were tagged with the date, which was considered day ‘0’ of anthesis for the superior spikelets. This process was repeated for the next three days. The spikelets on the basal primary branches reached anthesis 3–4 days later and were referred to as basal (inferior) spikelets; the date was considered day ‘0’ of anthesis for the inferior spikelets. The primary tillers were harvested for the collection of spikelets at various stages of anthesis, and the spikelets were sampled at 3-day intervals, beginning on day ‘0’. In Kharif season 2013, spikelets were harvested on 0, 3 and 6 days after anthesis (DAA); in the following season (Kharif season 2014), the harvesting was extended to 9 and 12 DAA. The superior and inferior spikelet samples collected from individual panicles were placed separately in 15-mL Falcon tubes and immersed in liquid N_2_. The frozen samples were stored at -80°C until analysis. Separate samples were collected for measuring ethylene production. SSH cDNA library preparation and experimental validation of the resulting libraries were performed using the samples collected during Kharif season 2013, and further follow-up experiments were conducted using the samples collected during Kharif season 2014.

### RNA isolation and cDNA preparation

Total RNA from the superior and inferior spikelets collected on each sampling day and stored frozen at -80°C was extracted using TRIzol (Invitrogen, Life Technologies) following the manufacturer’s instructions. The individual RNA pellets obtained were suspended in DEPC water. The quality and quantity of the individual RNA preparations were determined using a NanoDrop spectrophotometer. The RNA preparations were stored at -80°C until use. QuantiTect Reverse Transcription Kit (Qiagen), which provides an optimized mixture of random primers and oligo-dT and gDNA Wipeout Buffer, was used to convert the RNA to cDNA. All of the reverse-transcription reactions were performed according to the protocol outlined in the kit’s manual, in a total volume of 20 μL with 1 μg of gDNA-free total RNA.

To prepare the radiolabeled cDNA probe, total RNA was isolated from the superior and inferior spikelets sampled on 3 DAA. PolyATtract^®^ mRNA Isolation System I (Promega, USA) was used to purify mRNA from the total RNA isolated following the procedure outlined in the instruction manual. The Super SMART PCR cDNA synthesis kit (Clontech, Palo Alto, CA, USA) was used to reverse transcribe 2 μg of the purified mRNA to obtain double-stranded cDNA in a 20-μL reaction solution following the instructions provided in the manual.

### Construction of SSH cDNA libraries

Based on panicle morphology (compactness) and ethylene evolution (high evolution), SSH cDNA libraries were prepared for the rice cultivar Mahalaxmi using the spikelet samples collected on 3 DAA, the period of very active endosperm cell division and the beginning of grain filling [5]; the procedure described by Sahu and Shaw was followed [18]. Total RNA isolated from both the superior and inferior spikelets was simultaneously processed for mRNA purification and cDNA preparation. Double-stranded cDNAs prepared from the superior and inferior spikelets were separately digested with RsaI for 1.5 h to produce blunt ends, and the digested products were then extracted with phenol:chloroform:isoamyl alcohol (25:24:1). The resulting aqueous phase was extracted twice with chloroform:isoamyl alcohol (24:1) to obtain the digested cDNAs in the upper aqueous layer, which were then ethanol precipitated and resuspended in nuclease-free water (Promega, USA). The RsaI-digested cDNAs of the apical (superior) spikelets (RDcDNAAS), as well as those of the basal (inferior) spikelets (RDcDNABS), were divided into four equal portions; one portion of each was separately ligated to the 5’ end of adapter-1 (supplied in the SSH kit) overnight at 16°C to yield the products ASA1 and BSA1, representing the adapter-1-ligated blunt-end cDNA populations of the superior and inferior spikelets, respectively. Similarly, ligation of the 5’ end of adaptor-2R (supplied in the SSH kit) to another portion of RDcDNAAS and RDcDNABS generated the products AS2R and BS2R, representing the adapter-2R-ligated blunt-end cDNA populations of the superior and inferior spikelets, respectively. PCR amplification of the actin gene using a gene-specific reverse primer and an adapter-specific forward primer was performed to evaluate the ligation of both adaptors. The remaining portions of RDcDNAAS and RDcDNABS were used as the ‘Driver’.

Two SSH cDNA libraries were created. One represented the enriched population of overexpressed (and specifically expressed) transcripts in superior spikelets compared with inferior spikelets. For this library, ASA1 was ‘Tester-A’, AS2R was ‘Tester-B’, and RDcDNABS served as the ‘Driver’. The other SSH cDNA library represented the enriched population of overexpressed (and specifically expressed) transcripts in inferior spikelets compared with superior spikelets. This library was created by considering BSA1 and BS2R as ‘Tester-A’ and ‘Tester-B’, respectively, and RDcDNAAS as the ‘Driver’. For the construction of each library, two rounds of hybridization were performed, as outlined in the instruction manual. The resulting hybrid cDNAs in each library were amplified by two rounds of PCR (primary and secondary), each using different sets of adaptor-specific primers. Primary PCR was conducted with an initial denaturation at 94°C for 3 min followed by 27 cycles of 94°C for 30 s, 50°C for 30 s and 72°C for 45 s and a final incubation at 72°C for 10 min. The primary PCR product was termed either the apical-forward SSH cDNA library (AFL) or basal-reverse SSH cDNA library (BRL), as appropriate. Nested Primer 1 (NP1) and Nested Primer 2R (NP2R) were used for the secondary PCR, which was conducted for 20 cycles under the same conditions using the tenfold-diluted primary PCR product. To sequence the individual libraries, the secondary PCR products were purified using a NucleoSpin Extraction Kit (Clontech, Cat. No. 635961), cloned into the pGEM-T Easy Vector (Promega, USA) and then transformed into JM109 *E*. *coli* competent cells. The transformed bacteria for both AFL and BRL were plated separately on LB agar plates, which were then incubated at 37°C for 24 h to allow the development of blue and white colonies.

### Screening and authentication of SSH libraries

White bacterial colonies were randomly selected and grown in 100 μL of LB-amp medium in a standard 96-well plate at 37°C for at least 2 h with shaking. A PCR mix containing the nested primers (NP1 and NP2R) was prepared in a 96-well format, and a 1-μL sample of each individual bacterial colony was transferred to a corresponding individual well. PCR was performed at 94°C for 30 s followed by 23 cycles of 95°C for 10 s and 68°C for 3 min. An aliquot from each of the 60 PCR products was individually mixed well with an equal volume of 0.6 N NaOH, and 1.25 μL of each mixture was blotted on a nylon membrane (N^+^, Amersham Hybond) in a 10 x 6 format. Four such blots were prepared for each AFL and BRL sample. The blots were neutralized for 2–4 min in 0.5 M Tris-HCl (pH 7.5) and washed with H_2_O, and the DNA was cross-linked to the membrane using a UV crosslinker at 120 mJ. The secondary PCR products (100 ng, prepared fresh) of AFL and BRL were labeled separately with α-^32^P-dATP via random primer labeling (PCR-select screening kit, Clontech) according to the instruction manual and then purified using a Chroma Spin-100 column (Clontech). These radiolabeled secondary PCR products were then hybridized individually with both the AFL and BRL blots in 450 x 35-mm hybridization bottles according to the hybridization protocol outlined in the differential screening kit manual (Clontech).

After hybridization overnight at 72°C, the blots were washed for 20 min at 68°C with low-stringency washing solution (2X SSC/0.5% SDS) and then with high-stringency (0.2X SSC/0.5% SDS) washing solution. The washed blots were then exposed to X-ray film overnight with an intensifying screen at -70°C, and the exposed films were developed using an automatic Kodak X-ray film processor.

### Sequencing and functional categorization of cloned ESTs

The inserts in the individual white colonies were amplified by colony PCR using nested primers (NP1 and NP2R) in a standard 96-well plate, and the amplified fragments from the individual colonies were sequenced using an Applied Biosystems sequencing machine. The adaptor sequences were removed manually. Only ESTs (expressed sequence tags) that were at least approximately 100 bp long were considered for further analysis. The ESTs were assessed for homology using the BLASTN program (default) in the MSU (http://rice.plantbiology.msu.edu) and NCBI (http://www.ncbi.nlm.nih.gov) databases and grouped into singletons and contigs, which were termed unigenes. The MIPS (Munich Information for Protein Sequences) functional catalog, which was developed considering the functions of proteins in *Arabidopsis thaliana*, was used to group the unigenes into functional categories. *Arabidopsis* locus IDs were assigned to the individual unigenes based on their homology to *Arabidopsis* genes and then entered into the MIPS functional catalog to cluster the unigenes into different functional categories.

### Validation of the SSH cDNA libraries

To validate whether AFL and BRL do represent genes overexpressed in the superior and inferior spikelets, dot blots were prepared for AFL and BRL cDNA clones, as described above in “Screening and authentication of the SSH libraries”. The SMARTer cDNA synthesis kit (Clontech) was used to prepare cDNA from total RNA isolated from superior and inferior spikelets. This kit not only specifically reverse transcribes mRNA but also facilitates its amplification for a few cycles using a thermocycler. The amplified cDNA from the superior and inferior spikelets was labeled separately with α-^32^P-dATP by random primer labeling and then hybridized individually with both the AFL and BRL blots, as described above. The blots were washed and exposed, and the X-ray films were developed as described above. Experimental validation of the SSH cDNA libraries was also performed by RT-PCR, as described in the next section.

### Expression studies by RT-PCR and RT-qPCR

Expression studies of a few representative AFL and BRL genes relevant to the study’s objectives were performed by RT-PCR and RT-qPCR using the cDNA prepared with QuantiTect Reverse-Transcription Kit (Qiagen), as described above. Gene-specific primers not less than 20 nt long and with Tm values no less than 59.0°C were designed using the NCBI Primer Blast software (http://www.ncbi.nlm.nih.gov/tools/primer-blast). The designed primers (S2 Table) were specific for *Oryza sativa* (japonica cultivar-group) and amplified fragments of 70–200 bp. Prior to this step, the individual sequences of the japonica cultivar group were searched for their similarity with the indica cultivar group at the Ensembl Plants site (http://plants.ensembl.org/index.html), and to ensure that they would work in all cases, the primers were designed based on regions showing complete homology. The expression analyses were performed in a comparative manner: the expression of genes in the long-duration compact-panicle cultivar Mahalaxmi was compared with the expression levels observed in the long-duration lax-panicle cultivar Upahar, and the expression of genes in the medium-duration compact-panicle cultivar OR-1918 was compared with the levels observed in the medium-duration lax-panicle cultivar Lalat.

For RT-PCR, PCR amplification was performed with a DNA engine (Bio-Rad) thermocycler using 3 μL of the 10-fold-diluted cDNA as the template, GoTaq^®^ DNA Polymerase (Promega, M8291) and the PCR nucleotide mixture (Promega, C1141), according to the protocol detailed in the manual. The PCR program was set to 94°C for 3 min, followed by 30 cycles of 94°C for 1 min, 60°C for 1 min and 72°C for 1 min, with a final incubation at 72°C for 10 min. The amplified products (7 μL) were resolved on a 2.0% agarose gel and visualized by ethidium bromide staining and UV illumination. Actin was used as the loading control and reference. For mitochondrial genes, the 19S rRNA was used as the reference, and the PCR was conducted for only 25 cycles.

QuantiTect SYBR Green PCR Kit (Qiagen) was used for RT-qPCR with LightCycler^®^ 480 Real-Time PCR System (Roche), and the reactions were individually run in volumes of 25 μL in a 96-well plate, as detailed in the manual. Actin served as the reference for each cDNA preparation, with the exception of mitochondrial genes, for which 19S rRNA was considered the reference gene. The expression levels of individual genes in the superior and inferior spikelets are presented as fold changes according to Pfaffl [19].

### JC-1 fluorescence studies in caryopses

Caryopses at 12 DAA were dissected from the superior and inferior spikelets of the compact-panicle cultivar Mahalaxmi and the lax-panicle cultivar Upahar. Each caryopsis was cut individually into two halves and immersed in 2 μg L^-1^ JC-1 (5,5’,6,6’-tetrachloro-1,1’,3,3’-tetraethyl-benzimidazolycarbocyanine iodide) in 10 mM PBS (phosphate-buffered saline) for 25 min in the dark [20]. After washing twice for 10 min, the JC-1-impregnated caryopses were fixed in Carnoy’s solution for 48 h and then processed using a standard paraffin embedding procedure. Transverse sections (5 μm thick) of the caryopses were obtained using a microtome; the sections were mounted on glass slides and observed under a fluorescence microscope (Leica M205FA Microsystem, Germany) equipped with DSR-ET filter (excitation wavelength 530–560 nm) and CCD camera (Leica DFC450 C).

## Results

### Differential screening and validation of SSH cDNA libraries

**Fig 1:**
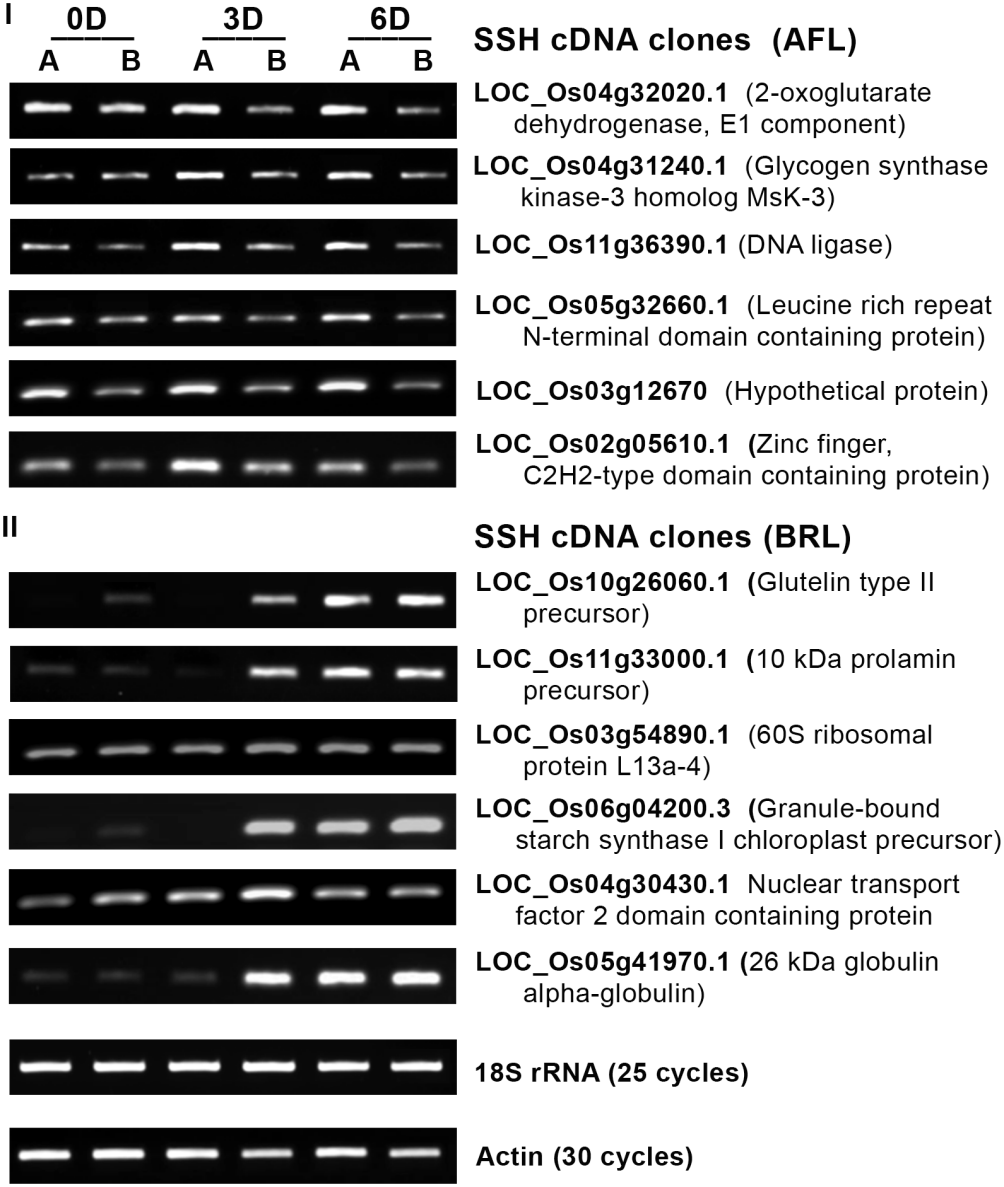
RT-PCR of total RNA isolated from superior (A) and inferior (B) spikelets of O. sativa cv. Mahalaxmi panicles on 0, 3 and 6 days after anthesis (DAA or simply D) for analysis of genes overexpressed in the superior compared with inferior spikelets (I) and genes overexpressed in the inferior compared with superior spikelets (II). Total RNA was isolated using TRIzol (Life Technologies) and reverse-transcribed using QuantiTect Reverse Transcription Kit (Qiagen). Each gene was amplified using gene-specific primers designed using Primer Blast. Actin and 18S rRNA were amplified for 30 and 25 cycles, respectively, as positive and loading controls. The other reactions were performed for 30 cycles, and the amplified products were separated on an agarose gel containing ETBR and visualized and photographed using a Gel Doc (Bio-Rad). The primer sequences are provided in S2 Table.

Differential screening revealed high-intensity signals upon hybridization of (1) the AFL blots with radiolabeled probes prepared either from the secondary PCR product of AFL or from the superior spikelet cDNA and of (2) the BRL blots with the probe prepared either from the secondary PCR product of BRL or from the inferior spikelet cDNA. These results confirmed the legitimacy of the libraries (S2 Fig). To further validate the SSH cDNA libraries, RT-PCR was performed for select genes that showed high EST abundance in AFL or BRL. The expression of the individual genes was studied at a spatiotemporal scale (Fig 1). As expected, all of the selected genes that showed high EST abundance in AFL were expressed at higher levels in superior compared with inferior spikelets at all of the tested time points (Fig 1I); similarly, those showing high EST abundance in BRL showed higher expression levels in inferior compared with superior spikelets (Fig 1II). Differences in the expression of genes encoding seed storage proteins, such as glutelin, prolamin and globulin, were found compared with other genes. Their expression began on 3 DAA in inferior spikelets and on 6 DAA in superior spikelets, after which similar expression levels were observed in both spikelets.

### Sequencing of SSH cDNA libraries and contig assembly

Table 1 summarizes the cloning and sequencing of AFL and BRL. More than 400 ESTs were sequenced for each library, and A BLASTN search of the ESTs revealed the presence of 57 unigenes (22 singletons and 35 contiguous sequences) in AFL and 79 unigenes (33 singletons and 46 contiguous sequences) in BRL. A contig EST redundancy analysis revealed that 94.80 and 92.48% of the genes in AFL and BRL, respectively, were sequenced.

**Table 1:** EST summary of the Apical-forward and Basal-reverse SSH cDNA libraries of O. sativa cv. Mahalaxmi prepared from RNA isolated from superior and inferior spikelets on 3 days after anthesis (DAA). The secondary PCR products of AFL and BRL were cloned, and the ESTs from these clones were sequenced.

Descriptive category	Apical-forward SSH cDNA library (AFL)	Basal-reverse SSH cDNA library (BRL)
Total ESTs sequenced	427	439
No. of singletons	22	33
No. of contiguous sequences (contigs)	35	46
No. of unigenes	57	79
No. of ESTs in contigs	405	406
Contig EST redundancy (%)^a^	94.80	92.48

^a^Percentage of the fraction of ESTs assembled in the contigs/total number of ESTs.

### Functional annotation of unigenes showing high EST redundancy

S3 Table represents the functional annotation of the genes present in AFL and BRL with EST redundancy values of at least two. Among the genes present in AFL, two encode proteins like beta-ketoacyl reductase and E1 component of 2-oxoglutarate dehydrogenase that function in the mitochondria, participating in fatty acid synthesis and the TCA cycle, respectively. Another gene encodes RFC1 protein required for DNA synthesis, whereas three genes encode proteins like LRR, GSK-3 and Znf having regulatory functions. In contrast, most of the genes present in BRL encode seed storage proteins, such as prolamin, glutelin and globulin. The gene with the highest EST redundancy in BRL encodes HMG1 protein, which supports transcription.

### Annotation and functional categorization of unigenes

The two SSH cDNA libraries (AFL and BRL) revealed highly functionally diverse sets of unigenes, as expected (S3 Fig). The contribution of the unigenes to different biological functions (S3A and S3B Fig) as well as the percentage of ESTs contributing to various functional categories (S3C and S3D Fig) differed between the two libraries. For example, 1) genes encoding seed storage proteins were not found in AFL (sub-category C, S3A Fig), whereas 1.1% of the unigenes fell in this category in BRL (S3B Fig); 2) 0.3% of the unigenes in AFL contribute to protein synthesis (F) compared with 4.3% in BRL (S3A Fig); 3) a greater number of unigenes in the Information category (sub-categories: Protein with Binding Function and Protein Activity Regulation, H and I, S3A Fig) were found in AFL compared with BRL, which did not reveal any unigenes contributing to protein activity regulation (I, S3B Fig); 4) 51.1% of the ESTs in BRL are categorized as genes encoding seed storage proteins (C, S3D Fig), versus none in AFL (S3C Fig); 5) 11.3% of the ESTs in BRL are involved in protein synthesis (F) compared with 0.8% in AFL (S3C and S3D Fig); 6) AFL showed 13.0% and 15.0% ESTs associated with the determination of cell fate (O) and organ localization (U), respectively (S3C Fig), but no such ESTs were present in BRL (S3D Fig); 7) In contrast to AFL, no ESTs contributing to tissue differentiation (R) and organ differentiation (S) were present in BRL (S3C and S3D Fig).

### Expression studies of differentially expressed unigenes across cultivars

**Fig 2:**
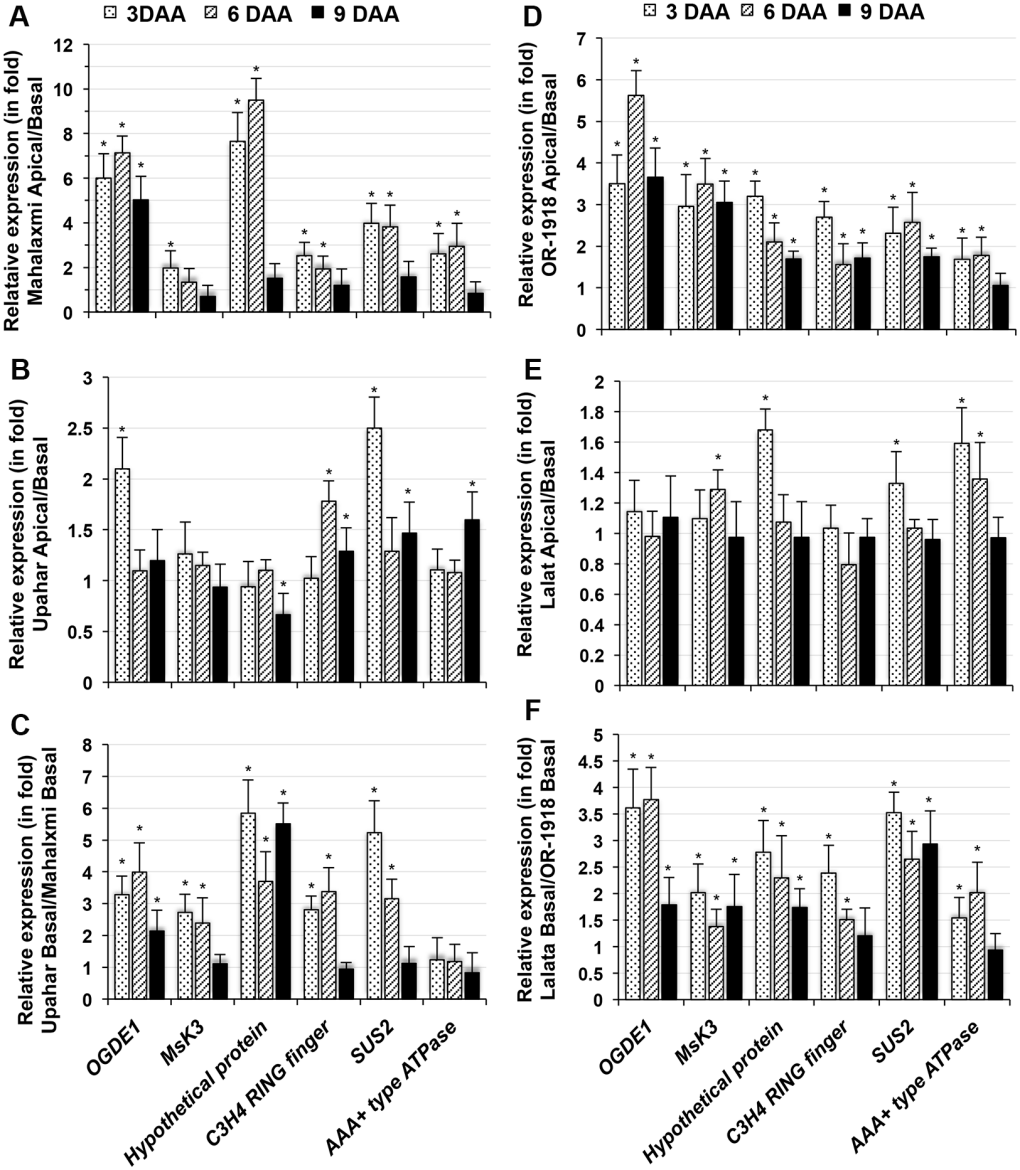
RT-qPCR of total RNA isolated from superior and inferior spikelets of *O*. *sativa* cultivars on various days after anthesis (DAA) for genes identified in the Apical-forward SSH cDNA library. Each bar represents the relative expression (in fold change) of a gene in superior compared with inferior spikelets in Mahalaxmi (A), Upahar (B), OR-1918 (D) and Lalat (E), in inferior spikelets of Upahar compared with inferior spikelets of Mahalaxmi (C) and in inferior spikelets of Lalat compared with inferior spikelets of OR-1918 (F). cDNA was prepared as described in Fig 1 and used as the template for RT-qPCR, which was conducted using QuantiFast SYBR Green PCR Kit (Qiagen) and a Roche LightCycler 480 thermocycler. Each gene, as well as actin as the reference control, was amplified using gene-specific primers (S2 Table) designed with Primer Blast. The fold change in expression was calculated by the ΔΔCt method with actin as the reference. Each bar represents the mean ± SD from three independent estimations. The asterisks (*) against each bar represent statistically significant changes in expression (either higher or lower in terms of fold change), at p ≤ 0.05, as determined by the ‘t’ test. The genes examined included 2-oxoglutarate dehydrogenase E1 component (*OGDE1*, LOC_OS04G32020.1), glycogen synthase kinase 3 (*GSK3*) *MsK3* homolog (LOC_OS04G31240.1), hypothetical protein (LOC_OS03G12670.1), sucrose synthase2 (*SUS2*, LOC_OS06G09450.2), AAA+ type ATPase (LOC_OS01G04814.1), and C3HC4 RING finger protein (LOC_OS07G31850.1).

The spatiotemporal expression of a few genes that presented high EST redundancy in either of the SSH cDNA libraries and/or that were important from the perspective of grain filling and metabolic regulation was analyzed in the rice cultivars Mahalaxmi, Upahar, OR-1918 and Lalat. In terms of maturity duration and panicle length, Mahalaxmi is similar to Upahar, and OR1918 is similar to Lalat (S1 Table). However, Mahalaxmi and OR-1918 are compact-panicle cultivars, whereas Upahar and Lalat are lax-panicle cultivars, as reflected by a small inter-grain space in the former compared with the latter (S1 Table). Moreover, grain filling is markedly lower in Mahalaxmi and OR-1918 than in Upahar and Lalat (S1 Table). Most of the genes selected from AFL, including 2-oxoglutarate dehydrogenase (*OGD*) E1 component (*OGDE1*), *MsK3*, C2H2 zinc-finger protein, sucrose synthase2 (*SUS2*), C3HC4 RING finger protein, AAA+-type ATPase, citrate transporter and a few hypothetical proteins, showed significantly higher expression in the superior compared with the inferior spikelets of Mahalaxmi and OR-1918 at least until 6 DAA (Figs 2A, 2D and S4). In lax-panicle Upahar and Lalat, however, these genes did not exhibit such differential expression (Fig 2B and 2E), with most showing relatively uniform expression throughout the observation period (S4 Fig). For some genes, such as sucrose synthase2 (LOC_OS06G09450.2) and AAA+-type ATPase (LOC_OS01G04814.1), expression declined with as the days after anthesis increased (S4 Fig). A comparative gene expression analysis of the two cultivars revealed significantly higher expression of all genes evaluated, with the exception of AAA+-type ATPase, in the inferior spikelets of Upahar and Lalat compared with those of Mahalaxmi and OR-1918 at least by 3 and 6 DAA (Fig 2C and 2F).

**Fig 3:**
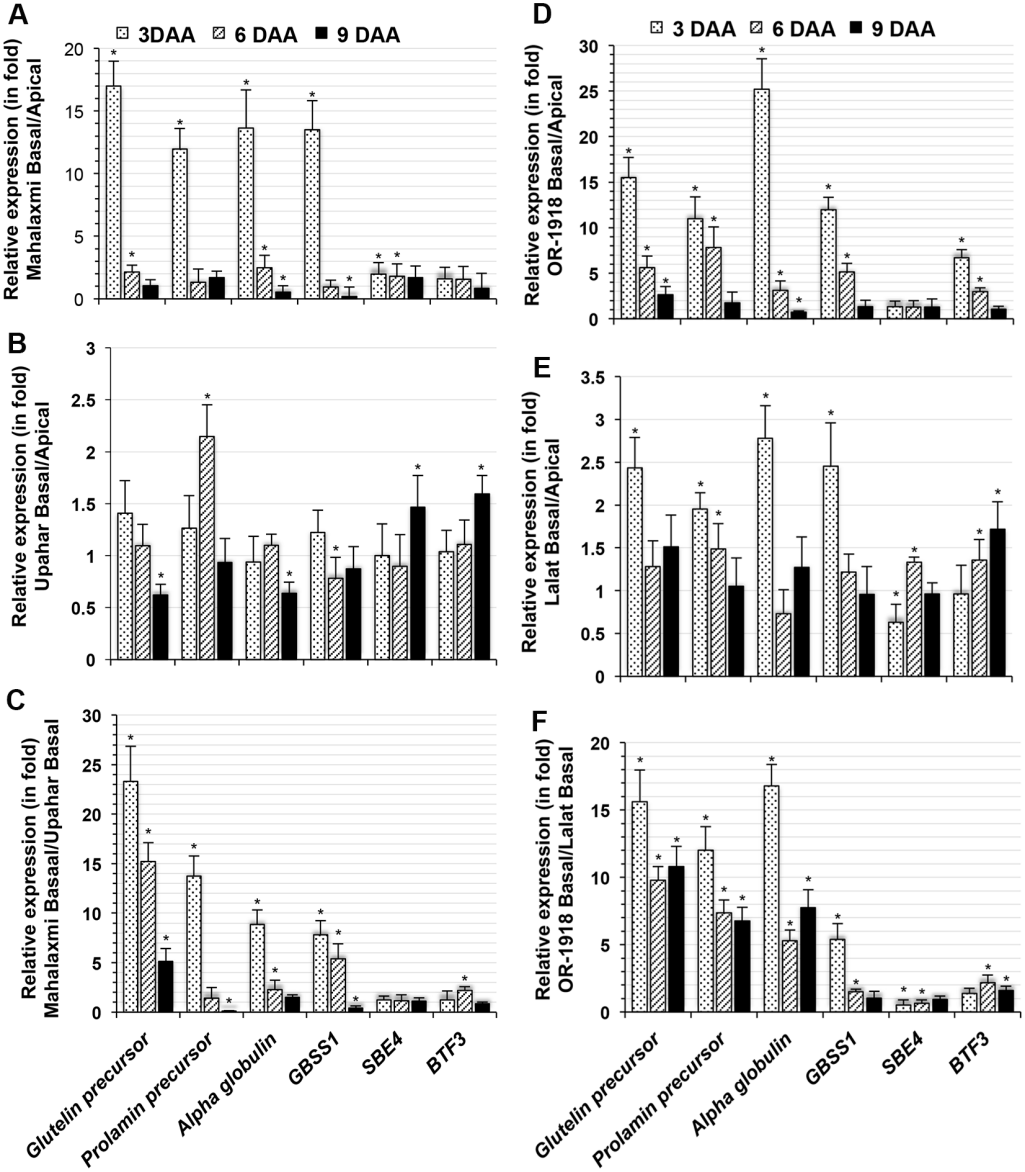
RT-qPCR of total RNA isolated from superior and inferior spikelets of *O*. *sativa* cultivars on various days after anthesis (DAA) for genes identified in the Basal-reverse SSH cDNA library. Each bar represents the relative expression (in fold changes) of a gene in inferior compared with superior spikelets in Mahalaxmi (A), Upahar (B), OR-1918 (D) and Lalat (E), in inferior spikelets of Mahalaxmi compared with inferior spikelets of Upahar (C) and in inferior spikelets of OR-1918 compared with inferior spikelets of Lalat (F). The genes examined included glutelin type II precursor (LOC_OS10G26060.1), prolamin precursor (LOC_OS11G33000.1), alpha-globulin (LOC_OS05G41970.1), granule bound starch synthase1 (*GBSS1*, LOC_OS06G04200.3), starch branching enzyme4 (*SBE4*, LOC_OS04G33460.1), and basal transcription factor3 (*BTF3*, LOC_OS03G01910.1). Additional details of the analysis are described in Fig 2.

Compared with superior spikelets, at least three storage proteins, glutelin type II precursor (LOC_OS10G26060.1), prolamin precursor (LOC_OS11G33000.1) and alpha-globulin (LOC_OS05G41970.1), and two genes, *GBSS1* (LOC_OS06G04200.3) and *SBE4* (LOC_OS04G33460.1), encoding enzymes involved in starch synthesis, were overexpressed in the inferior spikelets of Mahalaxmi (Figs 3A and S5). Similar to Mahalaxmi, OR-1918 also showed higher expression of most of these genes in inferior compared with superior spikelets at least by 3 DAA (Figs 3D and S5). Conversely, the expression of these genes in the inferior spikelets of the lax-panicle Upahar and Lalat were relatively similar to that in the superior spikelets (Figs 3B, 3E and S5), with the exception of *GBSS1*, which was significantly more highly expressed in inferior compared with superior spikelets on 3 DAA (Fig 3E). In addition, the inferior spikelets of Mahalaxmi and OR-1918 showed significantly higher constitutive expression of most genes compared with Upahar and Lalat, particularly on 3 and 6 DAA (Fig 3C and 3F).

### Expression studies of seed storage protein-associated transcription factors

**Fig 4:**
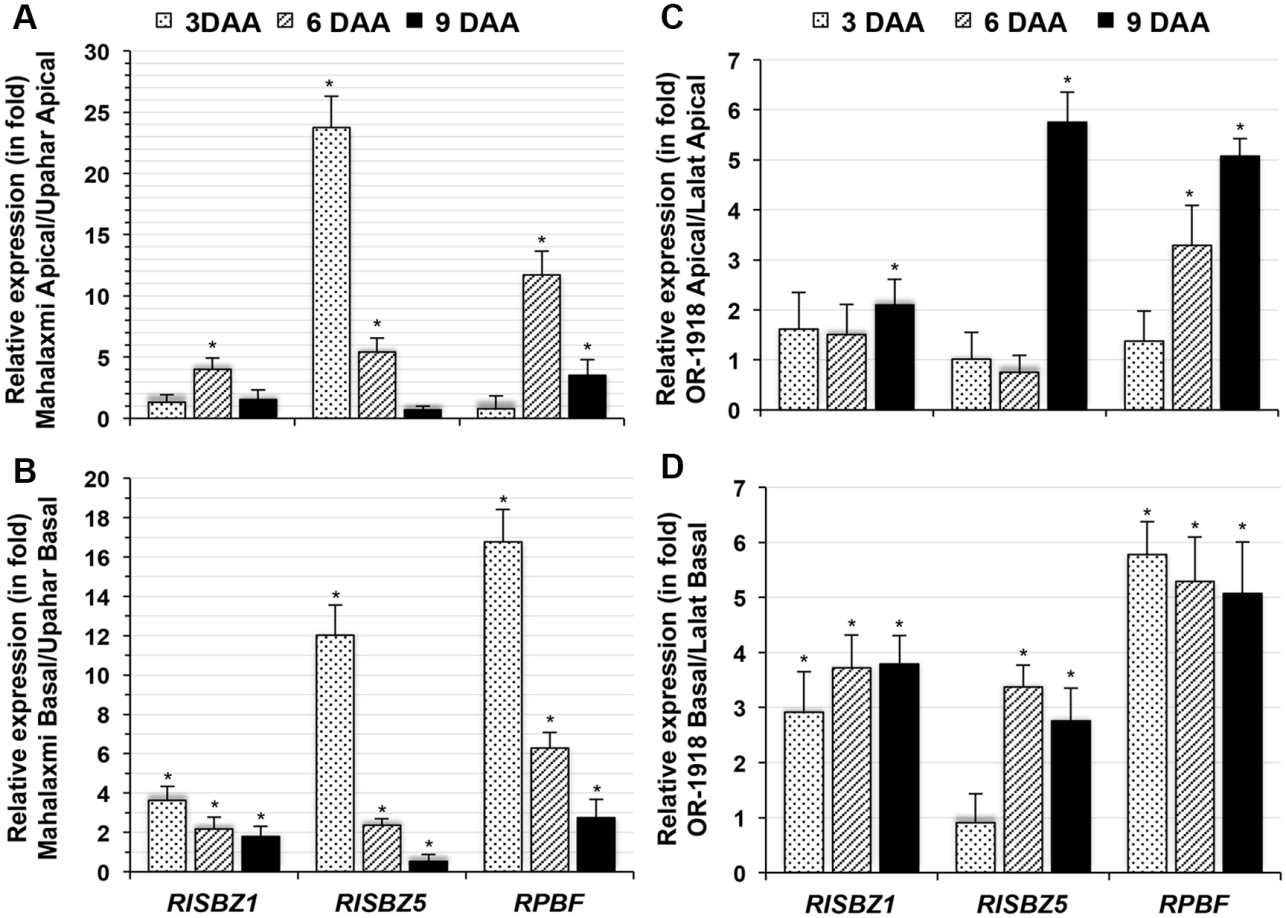
RT-qPCR of total RNA isolated from superior and inferior spikelets of *O*. *sativa* cultivars on various days after anthesis (DAA) for two isoforms of *RISBZ* (rice seed b-zipper) and *RPBF* (rice prolamin box-binding factor). Each bar represents the relative expression (in fold change) in superior (A) or inferior (B) spikelets of Mahalaxmi compared with those of Upahar and in superior (C) or inferior (D) spikelets of OR-1918 compared with those of Lalat. Additional details of the analysis are described in Fig 2.

The expression of genes encoding seed-storage proteins (SSPs) in rice is reportedly regulated by two proteins, RPBF (rice prolamin box binding factor, LOC_Os02g15350.1) and RISBZ (rice seed b-zipper), the latter of which has five isoforms (RISBZ1-5; LOC_Os07g08420.1, LOC_Os03g58250.1, LOC_Os02g16680.1, LOC_Os02g07840.1, LOC_Os06g45140.1). Analysis of the expression of these genes by RT-PCR revealed almost equal expression of *RISBZ2*, *RISBZ3* and *RISBZ4* on all of the sampling days in both Mahalaxmi and Upahar (S6 Fig). Only *RISBZ1* and *RISBZ5* showed low expression during the early days after anthesis compared with their expression on later days after anthesis (S6 Fig). Among these genes, the expression of *RISBZ5* was particularly low up to 3 DAA in Upahar and high during this period in Mahalaxmi in both superior and inferior spikelets (Fig 4A and 4B). *RISBZ1* exhibited comparatively lower expression in Upahar than in Mahalaxmi, particularly in inferior spikelets (Fig 4B), and the expression of this gene was particularly low in Upahar compared with Mahalaxmi on 3 DAA in inferior spikelets (Fig 4B) and on 6 DAA in superior spikelets (Fig 4A). In contrast to *RISBZ*, *RPBF* was expressed only after 3 DAA in Upahar, and the expression of this gene in Mahalaxmi was first detected in inferior spikelets on 3 DAA (S6 Fig). Furthermore, the constitutive expression of *RPBF* was markedly higher in Mahalaxmi than in Upahar, in both spikelets, and the difference was comparatively greater in the inferior compared with the superior spikelets (Fig 4A and 4B), particularly on 3 DAA (Fig 4B). Similar to its expression in Mahalaxmi, *RISBZ1* showed greater expression in inferior compared with superior spikelets on 3 DAA in OR-1918 (S6 Fig), though *RISBZ5* was not highly differentially expressed. The expression of *RPBF* in OR-1918 was also similar to that in Mahalaxmi (S6 Fig). RT-qPCR revealed that the constitutive expression of *RISBZ1*, *RISBZ5* and *RPBF* was markedly higher in OR-1918 than in Lalat in both spikelets (Fig 4C and 4D), similar to the results obtained from the Mahalaxmi and Upahar comparison (Fig 4A and 4B). However, *RISBZ5* exhibited markedly higher expression in OR-1918 than in Lalat in both spikelets on 9 DAA, resulting in a higher difference in expression (Fig 4C and 4D) than that observed in the comparison of Mahalaxmi with Upahar (Fig 4A and 4B).

### Expression studies of genes associated with the Krebs cycle

**Fig 5:**
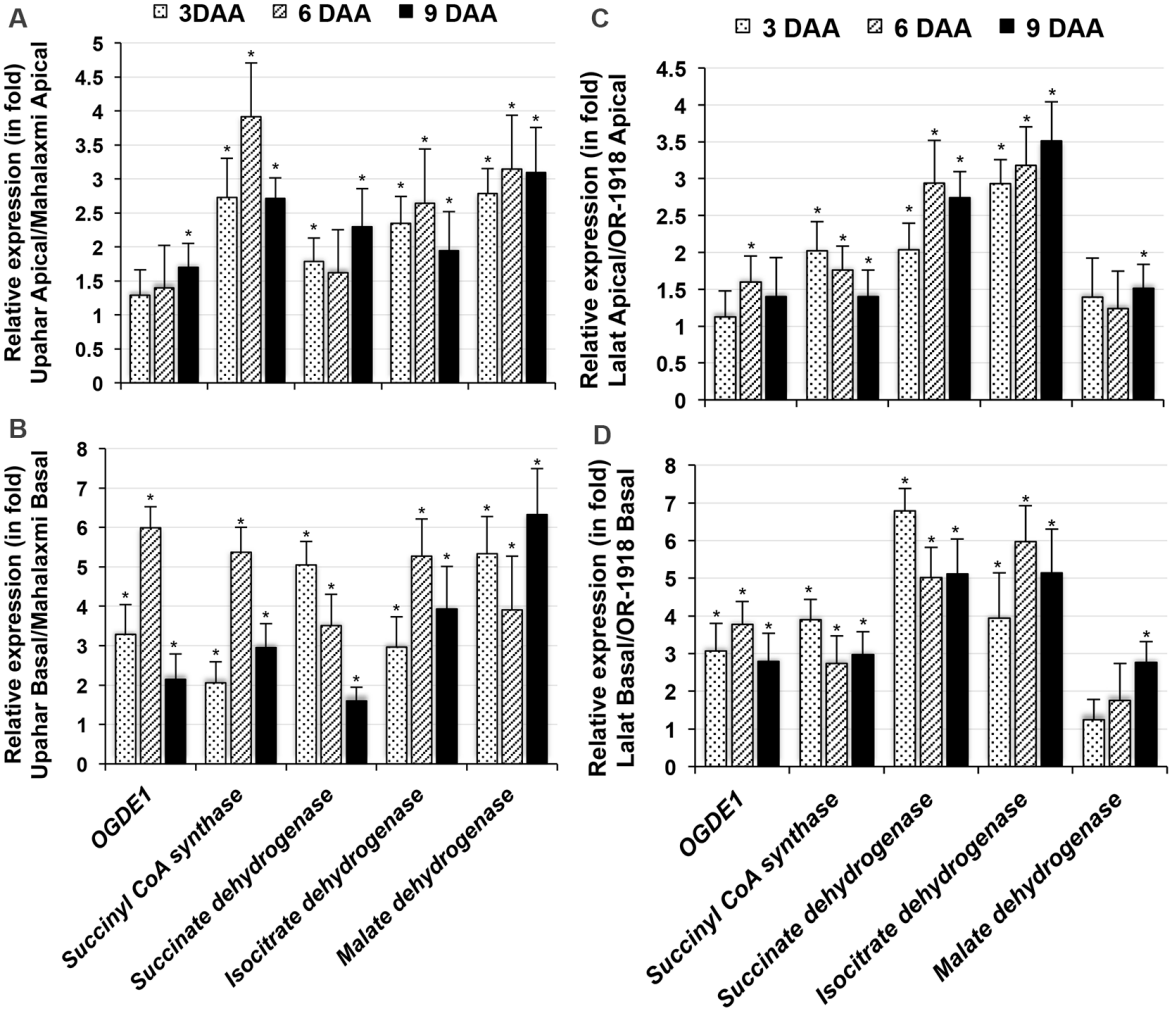
RT-qPCR of total RNA isolated from superior and inferior spikelets of *O*. *sativa* cultivars on various days after anthesis (DAA) for five mitochondrial genes. Each bar represents the relative expression (in fold change) in superior (A) or inferior (B) spikelets of Upahar compared with those of Mahalaxmi and in superior (C) inferior (D) spikelets of Lalat compared with those of OR-1918. 19S rRNA was used as the reference gene. The genes examined included 2-oxoglutarate dehydrogenase E1 component (*OGDE1*, LOC_OS04G32020.1), succinyl CoA synthase (LOC_Os07g38970.1), succinate dehydrogenase (LOC_OS07G04240.1), isocitrate dehydrogenase (LOC_Os01g46610.1), and malate dehydrogenase (LOC_Os01g46070.1). Additional details of the analysis are described in Fig 2.

The Krebs cycle-associated gene 2-oxoglutarate dehydrogenase E1 component (*ODGE1*, LOC_OS04G32020.1) was revealed in AFL, and the overexpression of this gene in superior compared with inferior spikelets of Mahalaxmi was confirmed by RT-PCR (S4 Fig). To explore the possible involvement of mitochondrial genes in the poor grain filling observed in compact-panicle rice cultivars, the spatiotemporal expression of selected genes of the Krebs cycle, namely, isocitrate dehydrogenase (LOC_Os01g46610.1), succinyl CoA synthase (LOC_Os07g38970.1), succinate dehydrogenase (LOC_OS07G04240.1) and malate dehydrogenase (LOC_Os01g46070.1), were studied in Mahalaxmi and the other three cultivars under study. Similar to *ODGE1*, these genes were also downregulated in inferior compared with superior spikelets of Mahalaxmi and OR-1918, at least by 3 and 6 DAA (S7 Fig). In contrast, the lax-panicle cultivars Upahar and Lalat displayed prominent and similar expression levels of all of these genes in both spikelets. The constitutive expression of all the genes in Upahar and Lalat was also greater than that in Mahalaxmi and OR-1918 and greater in inferior compared with superior spikelets (Fig 5A and 5D).

### JC-1 fluorescence in caryopses

**Fig 6:**
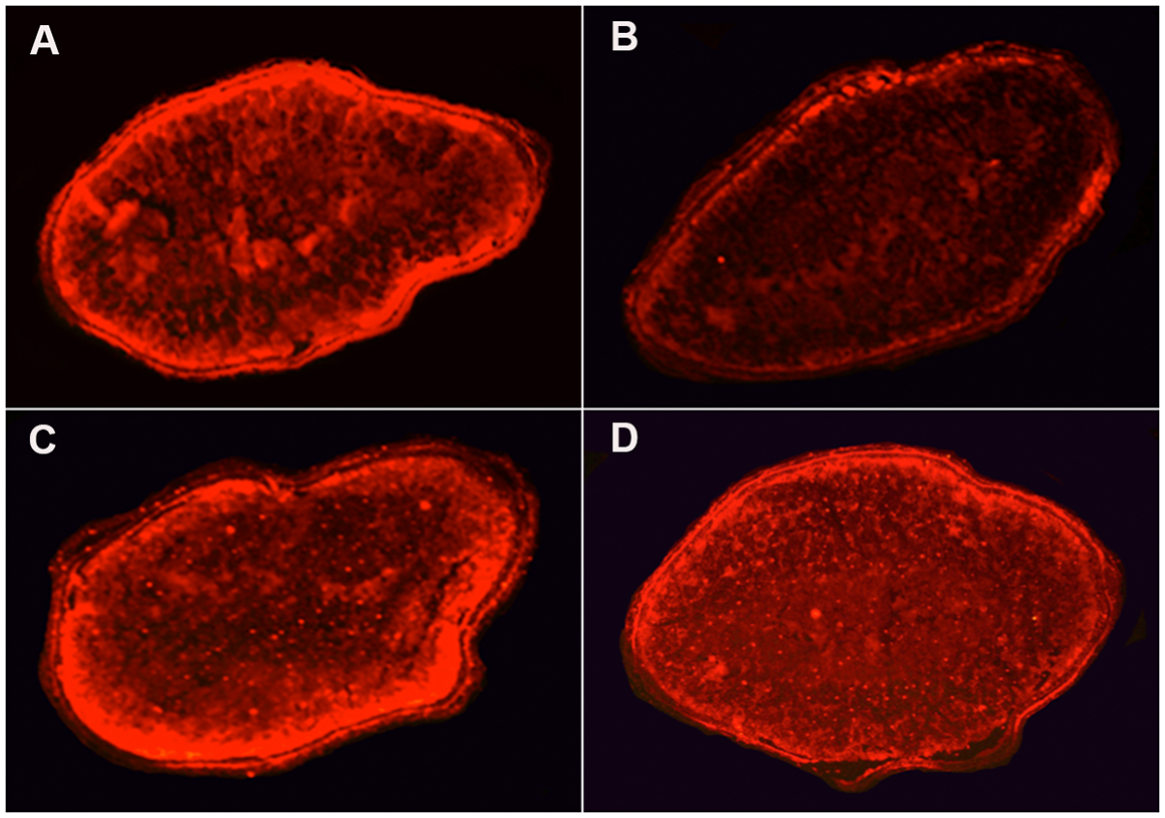
Transverse sections of caryopses from 12-DAA superior and inferior spikelets of the compact-panicle and lax-panicle rice cultivars Mahalaxmi and Upahar, respectively, showing JC-1 dye fluorescence.

The caryopses dissected from the superior and inferior spikelets of compact-panicle Mahalaxmi revealed differential JC-1 fluorescence: the fluorescence in the former was more intense than that in the latter (Fig 6). However, the fluorescence levels of JC-1 in caryopses from the superior and inferior spikelets of lax-panicle Upahar were essentially similar and comparable to that of the superior spikelets of Mahalaxmi.

## Discussion

It is quite established that in rice cultivars showing poor grain filling in inferior spikelets compared with superior ones, the backbone carbohydrate is not the limiting factor [6,21]. The differential grain filling observed in the inferior and superior spikelets of compact-panicle rice has been attributed to lower activities of starch-biosynthesizing enzymes in the former compared with the latter [8,17]. However, the real reason remains unclear. To date, differential proteomics and transcriptomics studies [21–23] have not been able to precisely address the issues of poor grain filling and poor-quality grain in inferior spikelets. Nevertheless, the importance of several genes in the grain filling process has been indicated, including that encoding the starch biosynthesizing enzymes, the enzymes mediating the respiratory process, the proteins/enzymes maintaining oxidoreductive homeostasis, and the proteins such as transcription factors and 14-3-3 proteins regulating biochemical processes at various levels [21–23].

Differential screening of the two SSH cDNA libraries using Apical-forward SSH cDNA and Basal-reverse SSH cDNA probes (S2A–S2D Fig) and virtual northern hybridization performed using Apical cDNA and Basal cDNA probes (S2E–S2H Fig) suggested that AFL and BRL represent genes overexpressed in the superior and inferior spikelets, respectively. RT-PCR conducted for selected genes from AFL and BRL at a temporal scale provide further evidence of the authenticity of the library preparation because these genes showed overexpression in the superior (Fig 1I) and inferior (Fig 1II) spikelets, respectively.

In addition to the differentially expressed genes identified (Table 1), the contig EST redundancy analysis revealed the possibility of identification of 5.20% and 7.52% additional unigenes in the AFL and BRL, respectively. One of the major differences noted between AFL and BRL was the presence of genes encoding seed storage proteins, which were only found in the latter (S3A and S3B Fig) and contributed 51.1% of the ESTs in the library (S3D Fig); this result indicated that excessive expression of these proteins in spikelets during the initial days of anthesis could be disadvantageous for the grain-filling process. Moreover, the poor contribution of genes encoding proteins with regulatory roles, such as proteins with binding functions (H), and the absence of unigenes encoding proteins involved in cellular communication (K), tissue differentiation (R), organ differentiation (S) and cell fate (O) in BRL compared with AFL suggest the failure of cellular regulatory and developmental processes as possible reasons for the poor grain filling observed in inferior spikelets.

The similarity in the spatiotemporal expression patterns of the unigenes in AFL, such as of *OGDE1*, *MsK*-3, C3HC4 RING finger proteins, *SUS2*, AAA+ protein and citrate transporter, in the spikelets of the compact-panicle rice cultivars Mahalaxmi and OR-1918 (S4 Fig) and, more importantly, their low expression in inferior compared with superior spikelets (Fig 2A and 2D) suggest that these genes may be involved in the grain-filling process and that their low expression results in poor grain filling in the inferior spikelets of compact-panicle rice cultivars. The importance of these genes in grain filling is further substantiated by the following observations: 1) these genes were expressed with high EST redundancy in superior spikelets (S3 Table); 2) there was no significant difference in their expression between the inferior and superior spikelets of the lax-panicle cultivars Upahar and Lalat (Fig 2B and 2E); and 3) the inferior spikelets of Upahar and Lalat showed significantly greater constitutive expression of these genes compared with that of Mahalaxmi and OR1918 (Fig 2C and 2F).

The biochemical and physiological roles of the above-listed genes have been studied in plants in some detail. 1) OGD primarily controls metabolic flux through the Krebs cycle, and overexpression of the E1 subunit fragment of the OGD complex in the antisense orientation in tomato (*Solanum lycopersicum*) considerably reduces the respiration rate in the tuber [24]. 2) MsK-3 is a homolog of glycogen synthase kinase-3 (GSK-3), a serine/threonine kinase that phosphorylates a wide range of substrates, including ATP citrate lyase, AMPc-dependent protein kinase, and transcription factors [25]. 3) The C3HC4 RING finger protein identified is similar to E3 ligases (XM_006658526.1), which are involved in the ubiquitination of target proteins, which leads to their proteasomal degradation and also regulates a variety of cellular processes, such as cell division, differentiation, signal transduction, and protein trafficking [26]. 4) Sucrose synthase (*SUS*) catalyzes the conversion of phloem sucrose to UDP-glucose, the first step in starch biosynthesis in the endosperm during grain filling [27]. 5) AAA and AAA+ (ATPases associated with diverse cellular activities) proteins represent a large class of Walker-type ATPases containing the AAA cassette that are functionally implicated in protein degradation as part of the 26S proteasome, the maturation of membrane complexes, gene expression, homo- and heterotypic membrane fusion and microtubule disassembly [28,29]. Considering the importance of biochemical processes such as ATP generation, protein modification and degradation and protein phosphorylation to cell growth and development and cell and tissue differentiation, it is plausible that low expression levels of genes regulating these biochemical processes, particularly the E1 subunit of the Krebs cycle OGD complex linked to ATP generation, would inhibit grain filling, as detected in inferior spikelets (Fig 2A and 2D). Moreover, the significantly higher expression of these genes in the inferior spikelets of lax-panicle cultivars compared with compact-panicle cultivars (Fig 2C and 2F) further substantiates the likelihood that their products must be essential for improved grain development. In addition, the reduced expression of *SUS* in inferior spikelets compared with superior spikelets in compact-panicle cultivars (Fig 2A and 2D) must be having a direct inhibitory effect on grain filling in the former. Regardless, the physiological and biochemical roles of hypothetical proteins in grain filling have not yet been explored.

Unlike most cereals, which utilize alcohol-soluble prolamins as a reserve, the major proteins present in rice seeds are alkaline-soluble glutelins, which may constitute up to 80% of the total endosperm proteins, with the prolamin fraction contributing only 5–10% [30]. The synthesis of these proteins is organ-specific, primarily regulated at the transcriptional level [31]. Duan and Sun [32] reported no detectable expression of glutelins and prolamins at 3 DAA, whereas in the current study, seed storage proteins were expressed from 3 DAA and thereafter in the compact-panicle cultivars (S5 Fig). The observation of very high expression levels of genes encoding seed storage proteins (10-kDa prolamin, glutelin and globulin) during the early period of grain filling in the inferior compared with the superior spikelets of compact-panicle cultivars (Fig 3A and 3D) suggests an inhibitory role of seed storage proteins on the grain filling process. The very low or no expression of seed storage protein genes prior to 6 DAA in the lax-panicle cultivars Upahar and Lalat (S5 Fig) and the very high constitutive expression of these genes in the inferior spikelets of the compact-panicle cultivars compared with that in the inferior spikelets of the lax-panicle cultivars (Fig 3C and 3F) indicated the same. However, the inhibitory effect of seed storage proteins on grain filling is yet to be explored.

Among the other genes overexpressed in Mahalaxmi inferior versus superior spikelets, any influence of *GBSS1*, *SBE4* and *BTF3* on the grain-filling process is unlikely because 1) higher constitutive expression of *GBSSI* was observed in the inferior spikelets of the compact-panicle rice cultivars compared with the inferior spikelets of the lax-panicle cultivars (Fig 3C and 3F), even though the former showed poorer grain filling; 2) *SBE4* expression did not differ significantly among the inferior spikelets of Upahar and Mahalaxmi (Fig 3C) although a significant difference in grain filling between the two cultivars was found (S1 Table); and 3) there was no difference in the constitutive expression of *BTF3* in the inferior spikelets of the lax- and compact-panicle cultivars (Fig 3C and 3F) despite the fact that grain filling differed significantly between the compact- and lax-panicle cultivars (S1 Table).

At least three of the five transcription factors, namely, *RISBZ1*, *RISBZ5* and *RPBF*, reported to regulate the expression of seed storage proteins [33,34] were found to be largely co-expressed with these proteins in all four rice cultivars (Figs 4 and S6) grown under similar conditions. This finding indicated that the expression of seed storage proteins in these cultivars is primarily regulated genetically. The results also suggested that the greater expression of *RPBF* in the inferior spikelets of compact-panicle cultivars compared with that in lax-panicle cultivars is the most likely reason for the higher expression of seed storage proteins in the former on 3 DAA (Fig 4). However, the basis for such cultivar-specific differences in the expression of either seed storage proteins or transcription factors remains unknown. Molecular marker-based phenotyping of these traits in a mapping population of a cross between compact-panicle and lax-panicle cultivars may provide the answer.

The grain-filling process has been reported to require the active participation of mitochondria as a source of energy [35,36]. Thus, because of the low expression of *OGDE1*, and consequent poor functioning of the Krebs cycle, mitochondria in the inferior spikelets of compact-panicle Mahalaxmi and OR-1918 (Fig 2A and 2D) may not be able to support the ATP requirements for the efficient transport of assimilates from the nucellus to endosperm cells and the biosynthesis of starch at a level similar to that in the superior spikelets, where the expression of this gene is comparatively high. This view is further substantiated by the following findings: first, Krebs cycle enzymes, including isocitrate dehydrogenase, succinyl CoA synthase, succinate dehydrogenase and malate dehydrogenase, whether operating downstream or upstream of ODG, exhibited lower expression in inferior compared with superior spikelets of the compact-panicle cultivars during most of the observation period, in contrast to their equally prominent expression in both superior and inferior spikelets of the lax-panicle cultivars (S7 Fig); second, the expression of the above-mentioned enzymes was significantly higher in spikelets of the lax-panicle cultivars than in those of the compact-panicle cultivars (Fig 5A–5D), and the lax-panicle to compact-panicle difference was greater for the inferior spikelets (Fig 5B and 5D) compared with the superior spikelets (Fig 5A and 5C). Poor ATP generation in the endosperm cells of the inferior spikelets of the compact-panicle cultivars compared with that in the superior spikelets of the compact-panicle cultivars and both spikelets of the lax-panicle cultivars is evident from the JC-1 staining of the caryopses. JC-1, a lipophilic membrane-permeable molecule that emits red fluorescence when the transmembrane potential of the mitochondria is highly negative, produced very intense fluorescence in the sections of caryopses from the superior and inferior spikelets of Upahar and of the superior spikelets of Mahalaxmi compared with the inferior spikelets of Mahalaxmi (Fig 6). The low negative transmembrane potential of mitochondria in the inferior spikelets of compact panicle cultivars, resulting in poor generation of ATP, is likely to be due to insufficient availability of the substrates for the mitochondrial electron transport chain because of poor functioning of the Krebs cycle.

## Conclusion

The results in this study indicated marked differences in metabolism between the superior and inferior spikelets of compact-panicle cultivars. The genes that promote grain filling may include the serine-threonine kinase *MsK3*, which coordinates cell cycle events, and the C3HC4 RING finger protein E3 ligase, which mediates protein modification and ubiquitination in a variety of cellular functions. Besides, the study indicated that poor grain filling is associated with high expression of seed storage proteins during the early days of grain filling. However, grain development may also remain poor if an active supply of assimilates from the nucellus to the aleurone layer and endosperm cells is not maintained and if the assimilates are not converted into starch due to poor generation of ATP, which occurs in the inferior spikelets of compact-panicle cultivars. Finally, examination of the complexities involved in grain development indicates that improvement in grain filling in the inferior spikelets of large compact-panicle rice cultivars is unlikely to be achieved by a breeding approach and that biotechnological interventions in this regard will also not be straightforward.

## Supporting Information

S1 Fig Ethylene evolution by the superior (apical) and inferior (basal) spikelets of the rice cultivars, including Mahalaxmi, Or-1918, TJ-112, Satyakrishna, Manika, Sebati, Upahar, Lalat, Ratna and Jaya. (PPTX)

S2 Fig Results of the differential screening of the clones from the Apical-forward and Basal-reverse SSH cDNA libraries. (PPTX)

S3 Fig Functional categorization of the unigenes overexpressed in the superior spikelets and inferior spikelets with respect to each other. (PPTX)

S4 Fig RT-PCR of total RNA isolated from the superior (A) and inferior (B) spikelets from panicles of *O*. *sativa* cultivars on various days after anthesis (DAA or simply D) showing amplification of the genes overexpressed in the superior compared to the inferior spikelets. (TIF)

S5 Fig RT-PCR of total RNA isolated from the superior (A) and inferior (B) spikelets from panicles of *O*. *sativa* cultivars on various days after anthesis (DAA or simply D) showing amplification of the genes overexpressed in the inferior compared to the superior spikelets. (TIF)

S6 Fig RT-PCR of total RNA isolated from the superior (A) and inferior (B) spikelets from panicles of *O*. *sativa* cultivars on various days after anthesis (DAA or simply D) showing amplification of various isoforms of *RISBZ* (rice seed b-Zipper) and *RPBF* (rice prolamin box-binding factor). (TIF)

S7 Fig RT-PCR of total RNA isolated from the superior (A) and inferior (B) spikelets from panicles of *O*. *sativa* cultivars on various days after anthesis (DAA or simply D) showing amplification of genes involved in the Krebs cycle encoding 2-oxoglutarate dehydrogenase, succinyl CoA synthase and succinate dehydrogenase. (TIF)

S1 Table Panicle morphology of the rice cultivars used in the study. (DOCX)

S2 Table Primers used in the study. (XLSX)

S3 Table Proteins with high EST redundancy (two or more) identified in the Apical-forward and Basal-reverse SSH cDNA libraries of *O*. *sativa* cv Mahalaxmi. (DOCX)
