# Supplementary material for: Identification and Characterization of Differentially Expressed Genes in Inferior and Superior Spikelets of Rice Cultivars with Contrasting Panicle-Compactness and Grain-Filling Properties
Source: PLoS One. 2015 Dec 28;10(12):e0145749. doi: 10.1371/journal.pone.0145749 (PMC4692420; doi:10.1371/journal.pone.0145749)
Supplement: S1 Fig — (PPTX) [file pone.0145749.s001.pptx]

## Slide 1
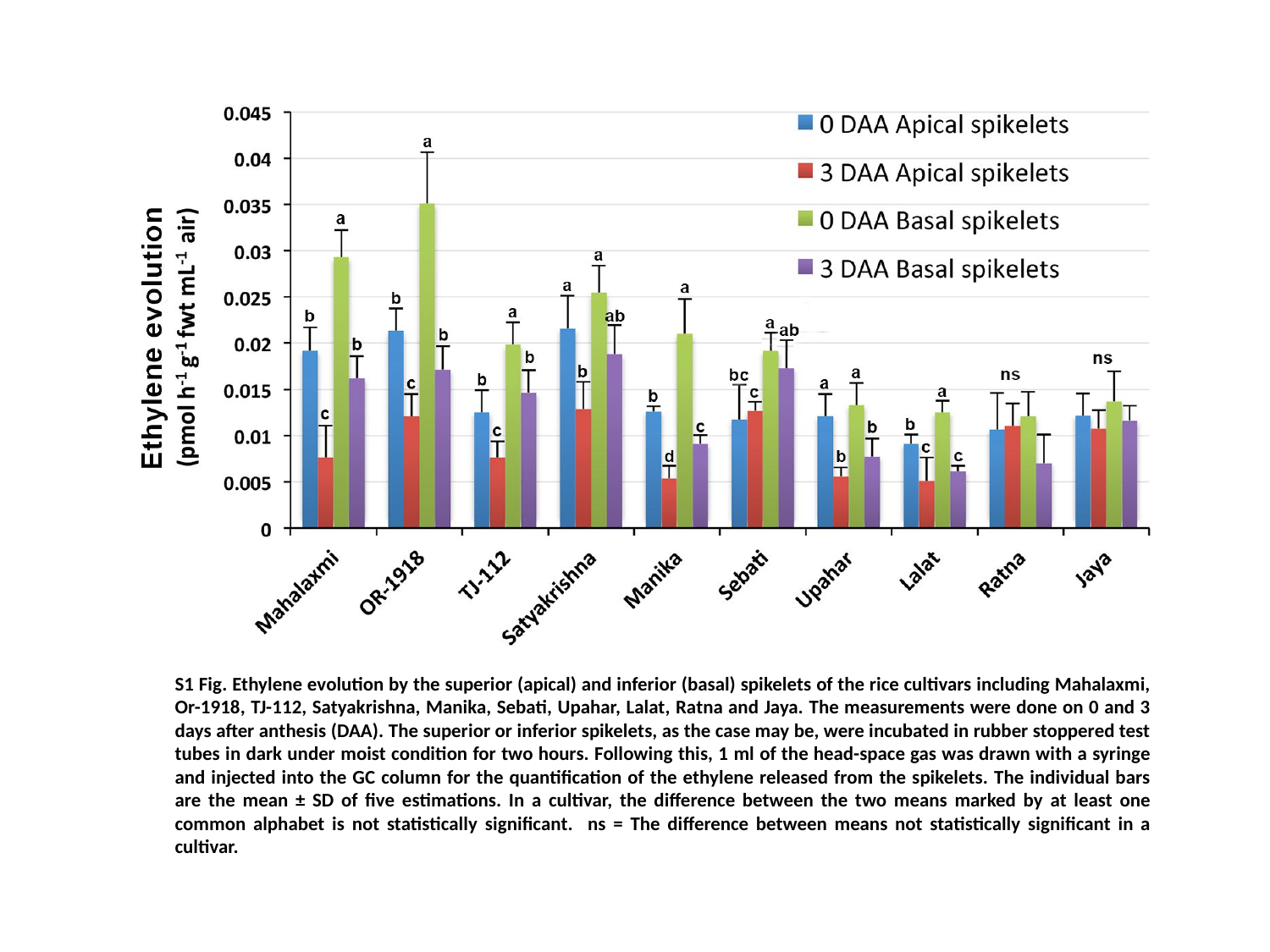

S1 Fig. Ethylene evolution by the superior (apical) and inferior (basal) spikelets of the rice cultivars including Mahalaxmi, Or-1918, TJ-112, Satyakrishna, Manika, Sebati, Upahar, Lalat, Ratna and Jaya. The measurements were done on 0 and 3 days after anthesis (DAA). The superior or inferior spikelets, as the case may be, were incubated in rubber stoppered test tubes in dark under moist condition for two hours. Following this, 1 ml of the head-space gas was drawn with a syringe and injected into the GC column for the quantification of the ethylene released from the spikelets. The individual bars are the mean ± SD of five estimations. In a cultivar, the difference between the two means marked by at least one common alphabet is not statistically significant. ns = The difference between means not statistically significant in a cultivar.
