# Supplementary material for: Identification and Characterization of Differentially Expressed Genes in Inferior and Superior Spikelets of Rice Cultivars with Contrasting Panicle-Compactness and Grain-Filling Properties
Source: PLoS One. 2015 Dec 28;10(12):e0145749. doi: 10.1371/journal.pone.0145749 (PMC4692420; doi:10.1371/journal.pone.0145749)
Supplement: S2 Fig — (PPTX) [file pone.0145749.s002.pptx]

## Slide 1
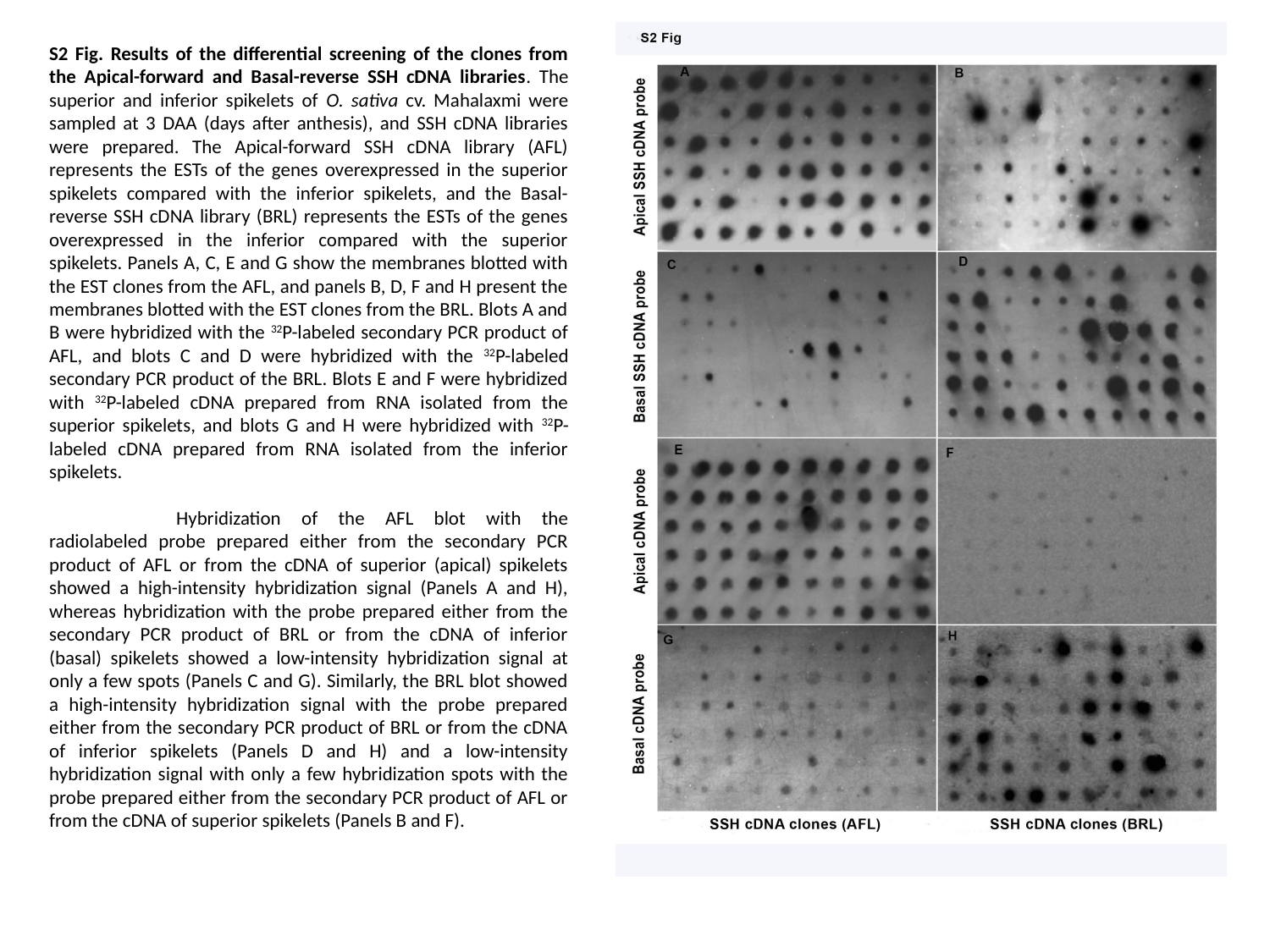

S2 Fig. Results of the differential screening of the clones from the Apical-forward and Basal-reverse SSH cDNA libraries. The superior and inferior spikelets of O. sativa cv. Mahalaxmi were sampled at 3 DAA (days after anthesis), and SSH cDNA libraries were prepared. The Apical-forward SSH cDNA library (AFL) represents the ESTs of the genes overexpressed in the superior spikelets compared with the inferior spikelets, and the Basal-reverse SSH cDNA library (BRL) represents the ESTs of the genes overexpressed in the inferior compared with the superior spikelets. Panels A, C, E and G show the membranes blotted with the EST clones from the AFL, and panels B, D, F and H present the membranes blotted with the EST clones from the BRL. Blots A and B were hybridized with the 32P-labeled secondary PCR product of AFL, and blots C and D were hybridized with the 32P-labeled secondary PCR product of the BRL. Blots E and F were hybridized with 32P-labeled cDNA prepared from RNA isolated from the superior spikelets, and blots G and H were hybridized with 32P-labeled cDNA prepared from RNA isolated from the inferior spikelets.
	Hybridization of the AFL blot with the radiolabeled probe prepared either from the secondary PCR product of AFL or from the cDNA of superior (apical) spikelets showed a high-intensity hybridization signal (Panels A and H), whereas hybridization with the probe prepared either from the secondary PCR product of BRL or from the cDNA of inferior (basal) spikelets showed a low-intensity hybridization signal at only a few spots (Panels C and G). Similarly, the BRL blot showed a high-intensity hybridization signal with the probe prepared either from the secondary PCR product of BRL or from the cDNA of inferior spikelets (Panels D and H) and a low-intensity hybridization signal with only a few hybridization spots with the probe prepared either from the secondary PCR product of AFL or from the cDNA of superior spikelets (Panels B and F).
