# Supplementary material for: Identification and Characterization of Differentially Expressed Genes in Inferior and Superior Spikelets of Rice Cultivars with Contrasting Panicle-Compactness and Grain-Filling Properties
Source: PLoS One. 2015 Dec 28;10(12):e0145749. doi: 10.1371/journal.pone.0145749 (PMC4692420; doi:10.1371/journal.pone.0145749)
Supplement: S3 Fig — (PPTX) [file pone.0145749.s003.pptx]

## Slide 1
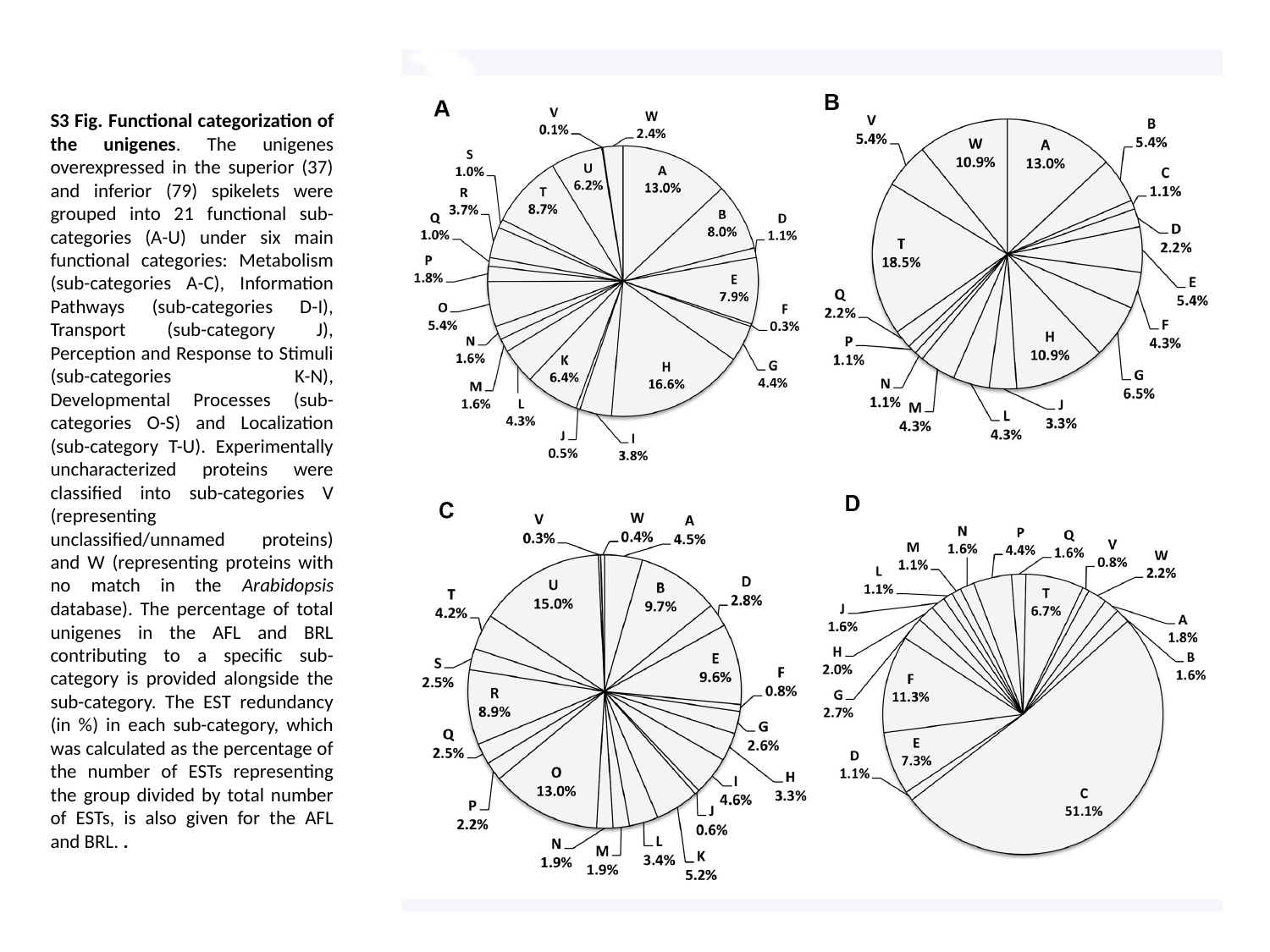

S3 Fig. Functional categorization of the unigenes. The unigenes overexpressed in the superior (37) and inferior (79) spikelets were grouped into 21 functional sub-categories (A-U) under six main functional categories: Metabolism (sub-categories A-C), Information Pathways (sub-categories D-I), Transport (sub-category J), Perception and Response to Stimuli (sub-categories K-N), Developmental Processes (sub-categories O-S) and Localization (sub-category T-U). Experimentally uncharacterized proteins were classified into sub-categories V (representing unclassified/unnamed proteins) and W (representing proteins with no match in the Arabidopsis database). The percentage of total unigenes in the AFL and BRL contributing to a specific sub-category is provided alongside the sub-category. The EST redundancy (in %) in each sub-category, which was calculated as the percentage of the number of ESTs representing the group divided by total number of ESTs, is also given for the AFL and BRL. .
