# Supplementary figures and images for: Identification and Characterization of Differentially Expressed Genes in Inferior and Superior Spikelets of Rice Cultivars with Contrasting Panicle-Compactness and Grain-Filling Properties
Source: PLoS One. 2015 Dec 28;10(12):e0145749. doi: 10.1371/journal.pone.0145749 (PMC4692420; doi:10.1371/journal.pone.0145749)

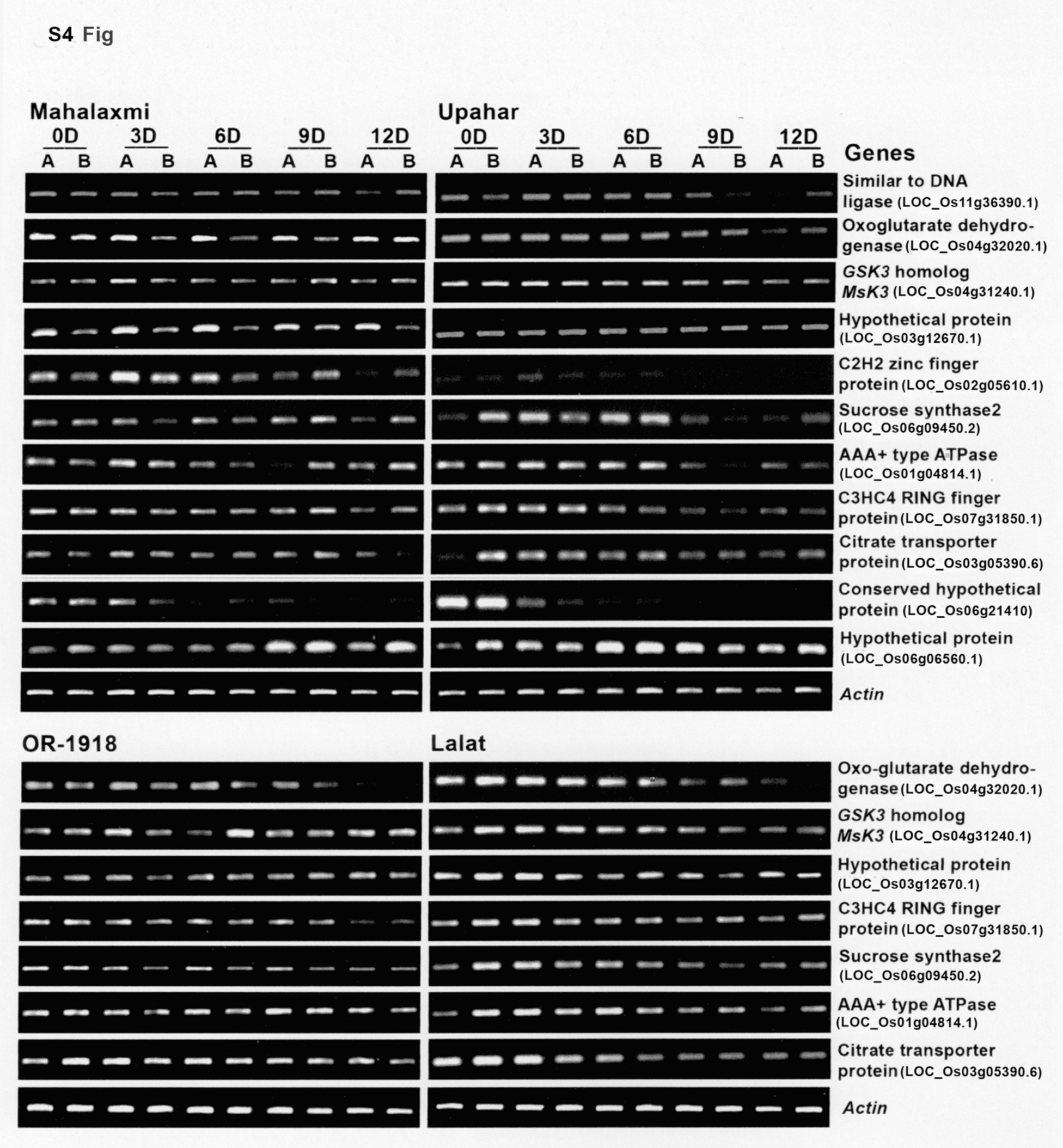

Supplement: S4 Fig — (TIF) [file pone.0145749.s004.tif]

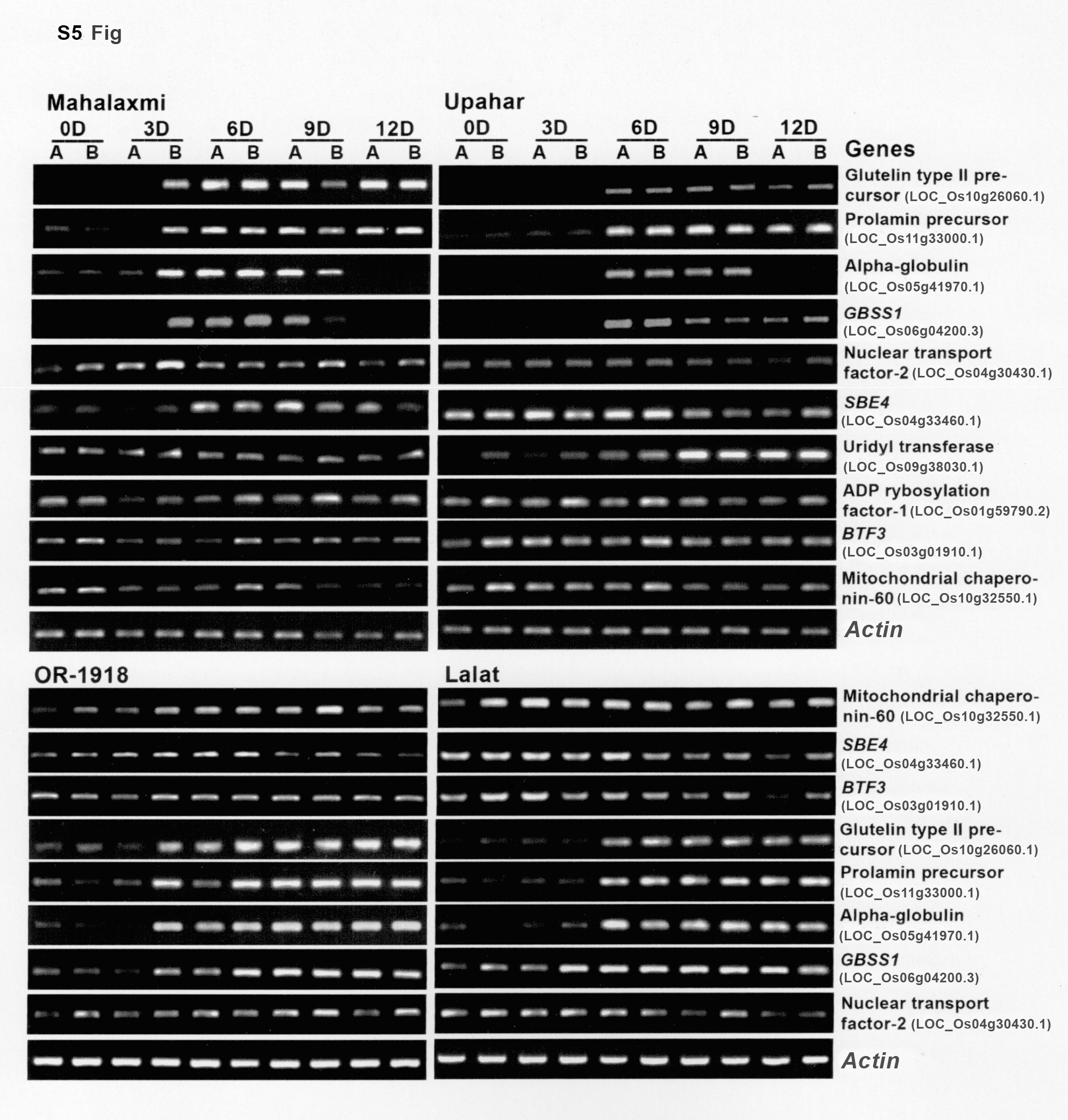

Supplement: S5 Fig — (TIF) [file pone.0145749.s005.tif]

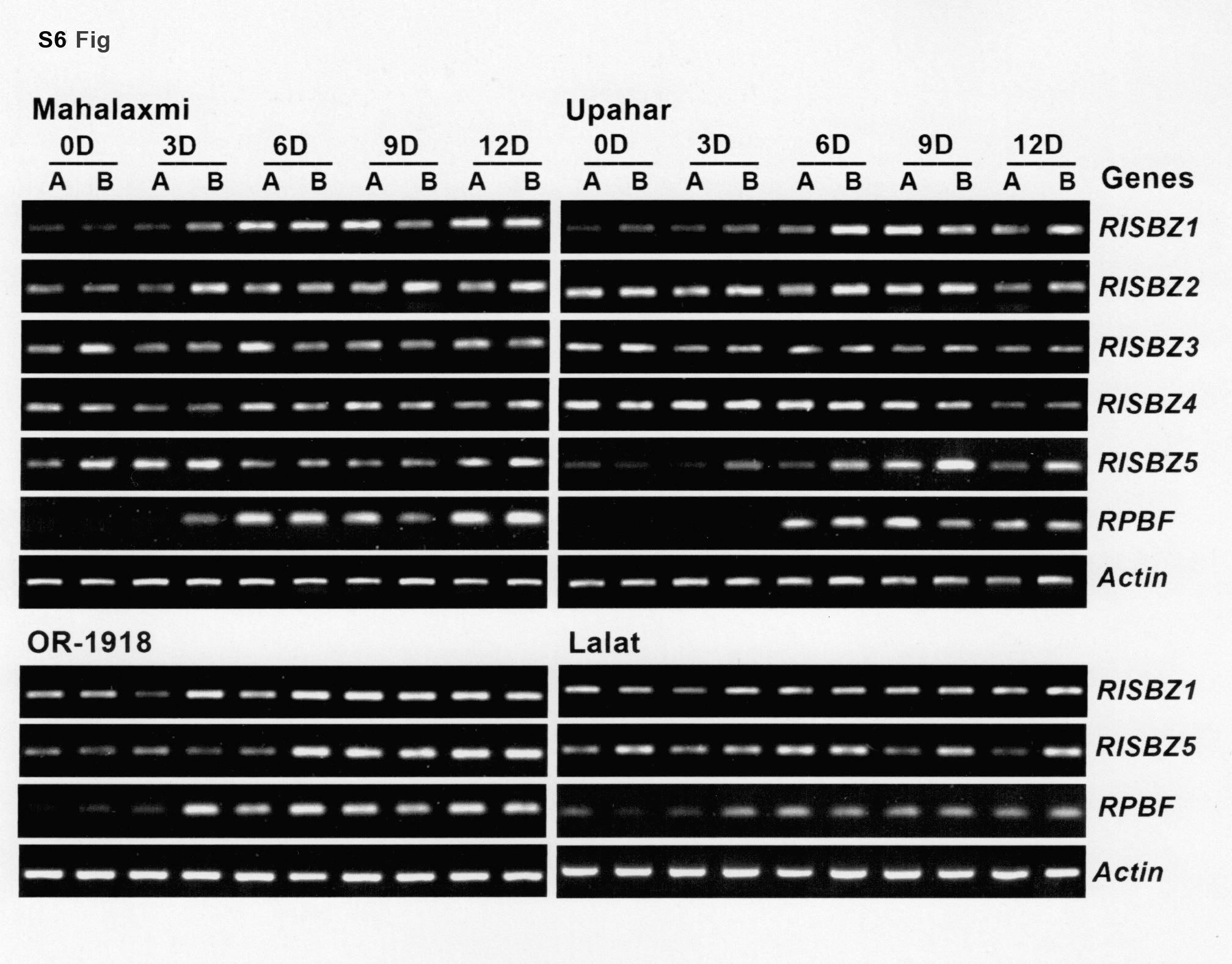

Supplement: S6 Fig — (TIF) [file pone.0145749.s006.tif]

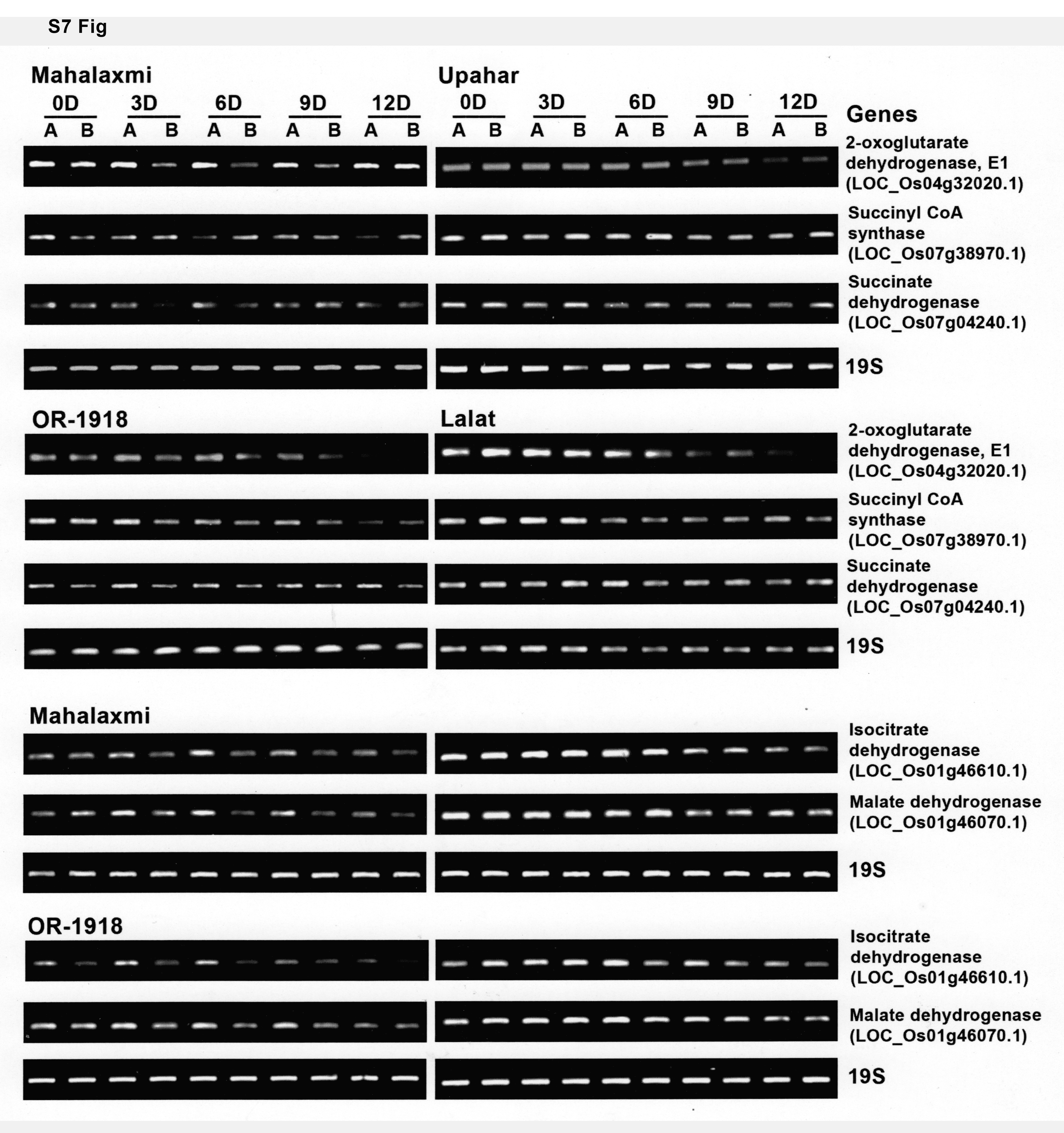

Supplement: S7 Fig — (TIF) [file pone.0145749.s007.tif]
