# Supplementary material for: Identification and Characterization of Differentially Expressed Genes in Inferior and Superior Spikelets of Rice Cultivars with Contrasting Panicle-Compactness and Grain-Filling Properties
Source: PLoS One. 2015 Dec 28;10(12):e0145749. doi: 10.1371/journal.pone.0145749 (PMC4692420; doi:10.1371/journal.pone.0145749)
Supplement: S1 Table — (DOCX) [file pone.0145749.s008.docx]

**S1 Table**. Morphological features of the panicle of main shoot of rice cultivars belonging to different duration groups at maturity. Cultivars Mahalaxmi, OR-1918, TJ-112, Satyakrishna, Sebati and Manika were compact-panicled and Upahar, Lalat, Ratna and Jaya were lax-panicled. Inter primary branch space = panicle length/number of primary branches per panicle, Inter-grain space = total length of panicle/grains per panicle. The values are means ± SD of five replicate studies. The means followed by common letters in superscript within a column shows no significant difference at (P < 0.05), as revealed by LSD (Least Significant Difference).

| **Cultivars** | **Duration of cultivars (Days)** | **Panicle length**  **(cm)** | **Total grain numbers** | **Filled grain (%)** | **Inter-grain space**  **(cm)** |
| --- | --- | --- | --- | --- | --- |
| **Upahar** | 140 | 30.95 ± 1.20^a^ | 221 ± 17.09^d^ | 91.55 ± 4.79^a^ | 0.140 ± 0.010^a^ |
| **Mahalaxmi** | 160 | 29.58 ± 1.95^a^ | 362 ± 10.59^a^ | 66.85 ± 2.00^d^ | 0.082 ± 0.006^d^ |
| **Lalat** | 125 | 26.75 ± 0.80^b^ | 212 ± 5.31^d^ | 88.88 ± 1.88^a^ | 0.126 ± 0.006^b^ |
| **OR 1918** | 110 | 22.45 ± 0.82^c^ | 255 ± 12.52^c^ | 55.00 ± 2.16^e^ | 0.088 ± 0.003^d^ |
| **Ratna** | 125 | 25.62 ± 1.15^b^ | 187 ± 3.65^e^ | 80.11 ± 5.16^b^ | 0.137 ± 0.003^a^ |
| **TJ112** | 150 | 22.37 ± 0.54^c^ | 274 ± 4.78^b^ | 72.56 ± 1.56^c^ | 0.082 ± 0.002^d^ |
| **Satyakrishna** | 135 | 25.75 ± 1.11^b^ | 289 ± 9.03^b^ | 72.97 ± 1.83^c^ | 0.088 ± 0.003^d^ |
| **Sebati** | 100 | 19.20 ± 0.51^d^ | 219 ± 5.29^d^ | 73.83 ± 1.33^c^ | 0.087 ± 0.006^d^ |
| **Jaya** | 135 | 27.12 ± 0.29^b^ | 218 ± 9.63^d^ | 80.00 ± 3.25^b^ | 0.124 ± 0.005^b^ |
| **Manika** | 165 | 23.30 ± 0.69^c^ | 232 ± 9.74^c^ | 86.92 ± 4.74^a^ | 0.100 ± 0.004^c^ |
| **5% LSD** |  | 1.82 | 16.24 | 5.82 | 0.009 |
