# Supplementary material for: Identification and Characterization of Differentially Expressed Genes in Inferior and Superior Spikelets of Rice Cultivars with Contrasting Panicle-Compactness and Grain-Filling Properties
Source: PLoS One. 2015 Dec 28;10(12):e0145749. doi: 10.1371/journal.pone.0145749 (PMC4692420; doi:10.1371/journal.pone.0145749)
Supplement: S3 Table — (DOCX) [file pone.0145749.s010.docx]

**S3 Table**. Proteins with high EST redundancy (two or more) identified in the Apical-forward and Basal-reverse SSH cDNA libraries of O. sativa cv Mahalaxmi. The libraries were prepared using RNA isolated from the superior (apical) and inferior (basal) spikelets on the 3 DAA day after anthesis. The cDNAs of the library were cloned and transformed, and the ESTs from the clones were sequenced. ^a^Percentage of the fraction of ESTs representing a unigene/total number of ESTs.

| GenBank accession | Name of the proteins/genes | EST redundancy (%)a | BLASTX search E-value | Biological function |
| --- | --- | --- | --- | --- |
| **Clones from Apical-Forward SSH cDNA library** | | | | |
| LOC_OS11G24484.1 | Beta-ketoacyl reductase 1 | 14.83 | 0.0 | An enzyme participating in fatty acid biosynthesis |
| LOC_OS09G25760.1 | Senescence-associated protein 5 | 12.91 | 0.0 | An integral component of the membrane with unknown function |
| LOC_OS04G32020.1 | 2-oxoglutarate dehydrogenase, E1 component | 12.36 | 1e-141 | A key enzyme of the TCA cycle that converts 2-oxoglutarate to succinyl-CoA |
| LOC_OS11G36390.1 | RFC1, the largest subunit of replication factor C | 6.04 | 0.0 | Required for DNA replication |
| LOC_OS05G32660.1 | Leucine-rich repeat (LRR) family protein | 5.76 | 0.0 | Provides a structural framework for the protein-protein interactions and has unknown function. |
| LOC_OS01G14860.3 | Glycogen synthase kinase-3 homolog MsK-3 | 5.49 | 0.0 | A serine/threonine kinase that phosphorylates a wide range of proteins |
| LOC_OS02G05610.1 | Zinc finger (Znf), C2H2-type domain containing protein | 2.47 | 0.0 | Znf-containing proteins function in gene transcription, translation, chromatin remodeling, etc. |
| LOC_Os09g03610.1 | RNA recognition motif (RRM) of rice flowering time control protein FCA | 2.19 | 5e-51 | Required for control of the flowering time |
| **Clones from Basal-Reverse SSH cDNA library** | | | | |
| LOC_OS06G51220.2 | HMG1 protein (HMGB1) | 16.57 | 1e-118 | Supports transcription by facilitating the binding of transcription factors and other proteins to DNA |
| LOC_OS11G33000.1 | 10-kDa prolamin precursor | 4.85 | 1e-144 | Seed storage protein that serves as a source of nitrogen, carbon and sulfur for the young developing seedling |
| LOC_OS01G55690.1 | Glutelin subunit | 3.43 | 0.0 | Seed-storage protein |
| LOC_OS02G15169.1 | Glutelin precursor | 2.28 | 0.0 | Seed-storage protein |
| LOC_OS11G33000.1 | 10-kDa prolamin precursor | 2.28 | 1e-132 | Seed-storage protein |
| LOC_OS03G54890.1 | 60S ribosomal protein L13a-4 | 2.0 | 1e-131 | Protein synthesis |
| LOC_OS04G30430.1 | Nuclear transport factor 2 (NTF2) family protein | 2.0 | 1e-23 | Facilitates protein transport into the nucleus |
| LOC_OS05G41970.1 | 26-kDa globulin | 3.14 | 1e-52 | Seed-storage protein |
